# The increase in cell death rates in caloric restricted cells of the yeast helicase mutant *rrm3* is Sir complex dependent

**DOI:** 10.1038/s41598-023-45125-z

**Published:** 2023-10-19

**Authors:** Andreas S. Ivessa, Sukhwinder Singh

**Affiliations:** 1 Department of Cell Biology and Molecular Medicine, Rutgers New Jersey Medical School, Rutgers Biomedical and Health Sciences, 185 South Orange Avenue, Newark, NJ 07101-1709 USA; 2 Pathology and Laboratory Medicine/Flow Cytometry and Immunology Core Laboratory, Rutgers New Jersey Medical School, Rutgers Biomedical and Health Sciences, 185 South Orange Avenue, Newark, NJ 07101-1709 USA

**Keywords:** Genomic instability, Apoptosis, DNA damage checkpoints, Chromosomes

## Abstract

Calorie restriction (CR), which is a reduction in calorie intake without malnutrition, usually extends lifespan and improves tissue integrity. This report focuses on the relationship between nuclear genomic instability and dietary-restriction and its effect on cell survival. We demonstrate that the cell survival rates of the genomic instability yeast mutant *rrm3* change under metabolic restricted conditions. Rrm3 is a DNA helicase, chromosomal replication slows (and potentially stalls) in its absence with increased rates at over 1400 natural pause sites including sites within ribosomal DNA and tRNA genes. Whereas *rrm3* mutant cells have lower cell death rates compared to wild type (WT) in growth medium containing normal glucose levels (i.e., 2%), under CR growth conditions cell death rates increase in the *rrm3* mutant to levels, which are higher than WT. The silent-information-regulatory (Sir) protein complex and mitochondrial oxidative stress are required for the increase in cell death rates in the *rrm3* mutant when cells are transferred from growth medium containing 2% glucose to CR-medium. The Rad53 checkpoint protein is highly phosphorylated in the *rrm3* mutant in response to genomic instability in growth medium containing 2% glucose. Under CR, Rad53 phosphorylation is largely reduced in the *rrm3* mutant in a Sir-complex dependent manner. Since CR is an adjuvant treatment during chemotherapy, which may target genomic instability in cancer cells, our studies may gain further insight into how these therapy strategies can be improved.

## Introduction

Calorie restriction (CR), which is a well-known metabolic intervention, is a reduction in calorie intake without malnutrition. CR usually extends the lifespan in many model organisms from yeast to primates and improves tissue integrity in higher eukaryotes^1–6^. CR has beneficial physiological effects on genome stability (e.g., preventing the shortening of telomeres or instability in ribosomal DNA (rDNA)); CR reduces also the effects of numerous diseases including obesity or predisposition and progression of cancer^3,5,7^.

However, under certain conditions and/or genotype settings CR may have adverse effects on cell survival. Some translational studies demonstrated that CR in the form of intermittent fasting or CR-mimetic drugs as adjuvant therapies enhance the efficacy of chemotherapy and reduce metastasis^5,8–20^. The combined action of the chemotherapeutic agents and the CR conditions lead to increased DNA breakage in cancer cells as it has been determined by the comet assay^15,21^. CR-mediated longevity has been shown to be genotype dependent in numerous organisms including yeast, flies and mice^5,22^. The underlying mechanisms of how maintenance of genome stability and metabolic state of a cell interact are still not well understood.

In this report we used the *rrm3* yeast mutant as a model system for nuclear genomic instability. Rrm3 is a DNA helicase^23^. In its absence, replication pauses (sometimes stalls) with increased rates at over 1400 distinct chromosomal sites (also called: replication-pause-sites (RPS))^23–27^. RPS include tRNA genes, centromeres, telomeres, silent replication origins and sites within the ribosomal DNA (rDNA)^23,26–33^. Usually these RPS are occupied by non-histone protein complexes, which may cause replication slowing/stalling^24,26,29,34^. Some of these sites were identified in chromosome translocations and numerous of these sites even superimpose with the locations of gamma-H2AX phosphorylation events which indicate the presence of single-stranded DNA leading potentially to double-strand DNA (dsDNA) breaks^23,26,27,31,33,35–39^. Although the exact function of Rrm3 is not known, it is proposed that during DNA replication Rrm3 transiently displaces the non-histone protein complexes, which are tightly bound to the RPS^24,28,40–43^. Rrm3 may also have a role in facilitating replication past highly-transcribed regions and in terminating DNA replication at natural RPS^24,25,44^. Although it appears that Rrm3 is acting only at specific chromosomal locations, this helicase migrates with the replication complex and affects replication of all yeast chromosomes (even in regions lacking RPS)^24,25,45^. The functions of Rrm3 are conserved in the fission yeast *S. pombe*, which is evolutionary closer to mammalian systems, and they overlap also with some functions of its yeast homolog Pif1^46–51^. Rrm3/Pif1 has a homolog protein in humans (human Pif1 (hPif1)) which affects telomeres^52,53^. There are reports that hPif1 is involved in tumor cell proliferation and it is proposed to be a cancer therapy target^54–57^. Thus, the Rrm3/Pif1 yeast studies may have relevance to understanding tumor initiation and progression.

Though *rrm3* mutant cells are viable, survival of the *rrm3* mutant cells is dependent on multiple checkpoint activities^26,58,59^. Examples are the intra S-phase checkpoint which includes Sgs1, the MRX complex, Srs2, Top3, Mec1, Mrc1 and Rad53. Rad53 is the mammalian CHK2 homolog and an essential serine/threonine kinase regulating checkpoint responses throughout the cell cycle. Rad53 is hyperphosphorylated in response to DNA damage^60,61^. Rad53 phosphorylation in *rrm3* mutant cells indicates DNA damage such as collapsed forks and/or DNA breakage. Rrm3 itself is also phosphorylated by Rad53 in response to replication fork stalling (induced by hydroxy urea, HU)^62^. Rrm3 is proposed to be required for the survival after replication fork breakage and for efficient sister chromatid exchange^63^. Rrm3p enriches at inducible DNA breaks (i.e., induced by an HO endonuclease) in a replication-dependent manner^63^. In a recent report, helicase activities of Rrm3 and Rad5 were demonstrated to interact genetically to prevent recombinogenic DNA lesions^40^.

There is a relationship between glucose restriction, repressive chromatin, and presence/activity of Rad53^64^. The Sir (*S*ilent *I*nformation *R*egulatory protein) complex plays a role in this relationship. The Sir complex consists of the Sir2/Sir3/Sir4 proteins^65^. It is mainly present in subtelomeric and telomeric regions, at the silent mating type loci *HML* and *HMR*, and at the rDNA locus (particularly Sir2) and mediates silencing of gene transcription at those locations. Sir2 is a NAD-dependent histone deacetylase which is required for heterochromatin formation at telomeres, the silent mating type loci *HML* and *HMR*, and at the rDNA locus^66,67^. The Sir complex is also recruited to dsDNA breaks and in addition the Sir2/Sir3 proteins can be present directly at euchromatic DNA replication origins^68–71^. CR-restricted growth conditions can promote rDNA repeat stability and extension of lifespan by Sir2-dependent and -independent pathways^3,72–76^. For example, CR greatly decreases RNA–DNA hybrid levels in the intergenic spacers of rDNA (DNA location between the 5S and 35S rDNA genes) in WT as well as *sir2* mutants^75^.

Here, we found that compared to wild-type (WT) cells, chromosomal DNA breakage is lower genome-wide in *rrm3* mutant cells, which are cultured in medium containing 2% glucose. However, DNA breakage rates in *rrm3* mutant cells increase in all these regions in growth conditions of CR (compared to *rrm3* mutant cells grown in 2% glucose). CR-*rrm3* mutant cells have increased cell death rates compared to *rrm3* cells grown in medium containing 2% glucose. This increase in cell death rates in the CR-*rrm3* cell is dependent on members of the Sir-complex (Sir2/3/4) as well as on mitochondrial reactive oxygen species (ROS). In addition, the Rad53 checkpoint protein is less (or not at all) phosphorylated in CR-*rrm3* mutant. In the absence of the Sir-complex, Rad53 phosphorylation is fully restored in the CR-*rrm3* mutant. Since cancer cells have genomic instability (like *rrm3* mutant cells), our studies should give further insight into how CR, which is a cancer-adjuvant therapy strategy, may help to battle cancer.

## Results

### In *rrm3* mutant cells genome-wide DNA breakage rates are lower than wild type under normal growth conditions (2% glucose), but increase after transfer to caloric-restricted (CR) growth medium

Previously we demonstrated that in the absence of the DNA helicase Rrm3, replication pauses/stalls at multiple sites within ribosomal DNA (rDNA) and at other regions in the yeast genome^23,26,27^. Because the increased pausing/stalling of replication forks may result in DNA breakage, we determined the natural chromosomal DNA breakage rates in these (and other) regions using the standard terminal deoxynucleotidyl transferase (TdT) assay combined with PCR (see “Methods” section; Supplement Fig. 1)^77^. To study the interaction between genome stability and metabolic state of a cell we applied caloric-restricted (CR) growth conditions.

We measured DNA breakage rates in wild-type (WT) and *rrm3* mutant yeast cells, which were grown either in regular, rich growth medium (YEPD, containing 2% glucose) or in CR growth medium (YEPD, containing 0.05% glucose) to an early-logarithmic growth phase (OD about 0.1; see also “Methods” section for growth conditions). Initially we determined DNA breakage rates in rDNA, which consists of a repeated chromosomal array (100–150 identical 9.1 kbp repeats on chromosome XII), but also extrachromosomal rDNA circles (ERCs) (Supplement Fig. 1C)^27,78,79^. Each rDNA repeat can be split into two about equal-sized 4.5 kbp DNA fragments by digestion of the rDNA with the restriction enzyme *Bgl*II (called Bgl A and B fragments; Supplement Fig. 1C)^78^. Whereas the Bgl A fragment does not contain any sites, which perturb rDNA replication, the Bgl B fragment contains the site for the replication fork barrier (RFB), which blocks rDNA replication unidirectional (Supplement Fig. 1C)^78,80^. In addition, the Bgl B fragment contains the 5S rDNA gene and a site, where rDNA replication can be initiated (called *ARS*, autonomous replicative sequence). Replication forks may pause at both the 5S rDNA genes and inactive replication origins, when these sites are replicated passively by forks moving through this region (Supplement Fig. 1C)^27,81^.

**Figure 1:**
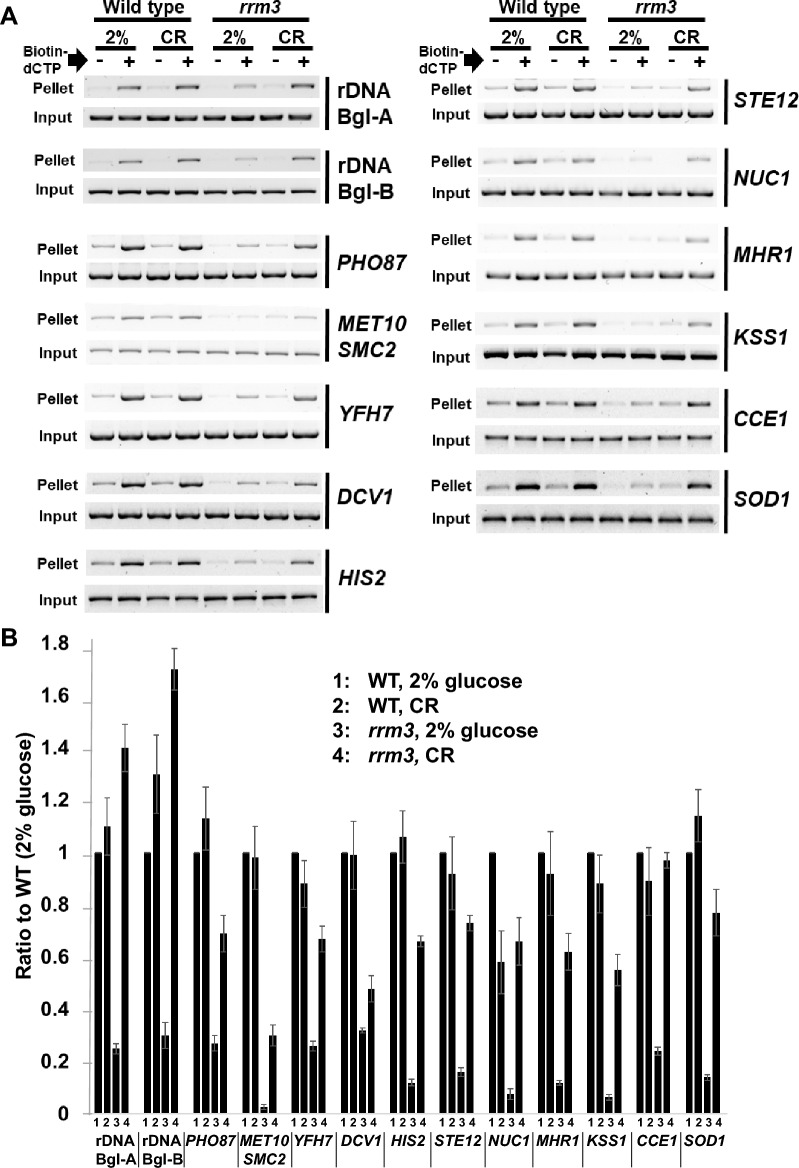
Genome-wide DNA breakage rates of *rrm3* mutant cells are low (compared to WT) under normal growth conditions (2% glucose) and increase after transferring to caloric-restricted (CR) growth conditions. (**A**) WT and *rrm3* mutant cells were grown in medium containing 2% or 0.05% (CR) glucose to an early logarithmic phase (OD_600_ ~ 0.1). Total genomic DNA was prepared, and DNA breakage rates were determined by using the terminal deoxynucleotidyl transferase (TdT) assay combined with PCR. Agarose gels display the PCR products for ribosomal DNA (rDNA) fragments #1 and #2 (see also Supplement Fig. 1C) and 11 indicated non-rDNA chromosomal locations. (−) and (+) biotin-dCTP refer to “no addition” or “addition” of biotin-dCTP to the TdT reaction. In the positive-control experiment, we demonstrate that the TdT assay can readily detect dsDNA breakage events, since genomic DNA which has been digested with a restriction enzyme *prior* to carrying out the TdT assay, results into higher DNA breakage rates (Supplement Fig. 1B). The full-sized images (pellet) are shown as Supplement Figures. (**B**) The intensity of the bands in the gels shown in panel A was determined by using the program ImageLab (Bio-Rad™ Laboratories). Each pellet value was first normalized by the input value, then the (−)-biotin-dCTP value (i.e., background) was subtracted from the (+)-biotin-dCTP value. The DNA breakage value for WT (2% glucose) was set at the arbitrary number 1. Experiments were carried out three times. Standard errors are shown.

Using the TdT assay, we demonstrate that natural DNA breakage rates are consistently three–fourfold lower in both rDNA fragments (Bgl A and B) of the *rrm3* mutant compared to WT cells (both grown in 2% glucose) (Fig. 1, rDNA Bgl A and B). WT cells experienced about 15% higher DNA breakage rates in medium containing 0.05% glucose (CR) compared to medium containing 2% glucose. DNA breakage rates in both rDNA fragments (Bgl A and B) of CR *rrm3* mutant cells increased on average 5.7-fold compared to *rrm3* mutant cells grown in 2% glucose. DNA breakage rates in rDNA of the CR-*rrm3* mutant cells are 40–80% higher than DNA breakage rates of WT-cells (2% glucose) in both Bgl A and B fragments.

To investigate whether in the *rrm3* mutant the low DNA breakage rates (compared to WT) and their increase after the switch from regular growth (2% glucose) to CR-growth conditions are specific for rDNA, we determined DNA breakage rates of several non-rDNA chromosomal regions. Some of these regions contain known (or putative) RPS (e.g., tRNA genes and silent replication origins). As described above, we determined DNA breakage rates by using the TdT assay (see Supplement Fig. 1). DNA was digested with the restriction enzyme *Bgl*II prior to the capture of the broken DNA fragments (having biotinylated extensions) with avidin-coated beads. DNA breakage rates were determined for the following eleven regions; the names of the probes (gene names) are listed as well as the sizes of the (unbroken) *Bgl*II fragments are indicated in parenthesis: *PHO87* (~ 23 kbp), *MET10*/*SMC2* (~ 2.1 kbp), *YFH7* (~ 6.2 kbp), *DCV1* (~ 9.2 kbp), *HIS2* (~ 5.3 kbp), *STE12* (~ 6 kbp), *NUC1* (~ 3 kbp), *MHR1* (~ 5 kbp), *KSS1* (~ 3 kbp), *CCE1* (~ 20 kbp) and *SOD1* (~ 10 kbp) (Fig. 1). Some of the investigated DNA fragments contain known (or putative) RPS. These fragments contain known (or putative) silent replication origins (in parenthesis): *PHO87* (*ARS313/314*), *MET10*/*SMC2* (*ARS608*), *KSS1* (*ARS723*) and *CCE1* (*ARS1113*)^26,28^. These fragments contain tRNA genes (in parenthesis): *YFH7* (*tRNA*^F^), *DCV1* (*tRNA*^Y^), *HIS2* (*tRNA*^A^) and *SOD1* (*tRNA*^L^)^26^.

In summary, the natural nuclear DNA breakage rates follow similar trends as observed for rDNA, though there are a few differences: (i) in average, about tenfold reduced DNA breakage rates in the *rrm3* mutant compared to WT cells (both 2% glucose), the presence of natural RPS (e.g., tRNA genes and silent replication origins) does not appear to have an effect on the changes in the DNA breakage rates; (ii) an in average 5.4-fold increase in DNA breakage rates in the *rrm3* mutant when cells are switched from regular growth medium (2% glucose) to CR-growth medium (0.05% glucose), however (unlike in rDNA) DNA breakage rates do not reach WT-levels (except for the DNA fragment containing *CCE1*); (iii) the breakage rates in chromosomal DNA of CR-WT cells are in average 5% increased or decreased compared to WT cells grown in medium containing 2% glucose (Fig. 1).

### In caloric-restricted (CR) *rrm3* mutant cells replication pausing/stalling is decreased at selective sites in ribosomal DNA, at tRNA genes and silent replication origins, but high-molecular weight DNA structures increase in ribosomal DNA

**Figure 2:**
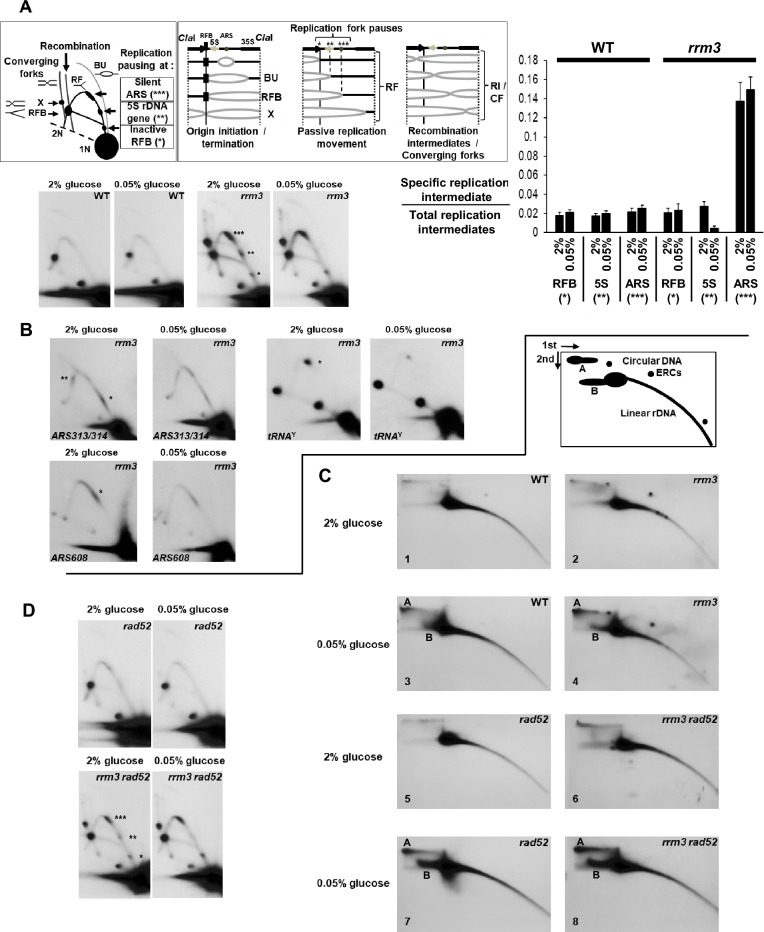
Replication pausing/stalling is decreased at sites in ribosomal DNA, at tRNA genes and silent replication origins of caloric-restricted *rrm3* mutant cells, but high-molecular weight DNA structures increase in ribosomal DNA under CR-growth conditions. (**A**) rDNA replication is initiated in a subset of the rDNA repeats. The pattern of replication intermediates for any rDNA fragment containing a replication origin (*ARS*: *a*utonomous *r*eplicative *s*equence) is a composite of intermediates generated from repeats with an active *ARS* and those without an active *ARS*^27,78^. For repeats with an active *ARS*, initiation begins and proceeds bi-directionally from the *ARS* (bubble structures, BU). They are converted to simple Y structures when the rightward-moving fork reaches the end of the fragment. The leftward-moving fork stops at the site-specific replication fork barrier (RFB) creating increased hybridization on the arc of simple Y-shaped intermediates. The remaining part of *Cla*I fragments with active *ARS*s is replicated by forks converging on the fork stalled at the RFB. These forks generate intermediates that emanate from the RFB and terminate with a mass of close to 2N (X). Repeats without an active origin are replicated uni-directionally, creating a full arc of simple Y-shaped intermediates (passive replication movement). Schema of rDNA replication intermediates (*Cla*I DNA fragment): *BU* bubble arc (i.e., replication initiation); *RFB* replication fork barrier; *X* termination of rDNA replication; *RF* replication forks; RI, recombination intermediates; *CF* converging forks. Pauses of replication forks at the natural RPS in rDNA are indicated (asterisks). A *Stu*I-*Bgl*II fragment within the analyzed *Cla*I-rDNA fragment served as a probe^81^. 2D gels of *Cla*I-digested DNA of the indicated strains and growth conditions are displayed. Probe: rDNA (*Stu*I-*Bgl*II). Histogram shows quantifications by phosphor-imager analysis. Pauses of replication forks at the natural RPS in rDNA are indicated by asterisks (*, **, ***). RFB refers to the in-active replication fork barrier (*); 5S means 5S rDNA gene (**); *ARS* refers to the silent replication origin (***). The amount of each replication intermediate is expressed as ratios (i.e., specific intermediate *versus* total replication structures (i.e., sum of all replication forks (including pauses and RFB), RI/CF, and BU). In this way, we normalized for different amounts of total replication structures. Experiments were carried out two times. Standard errors are displayed. (**B**) 2D gels of *Eco*RV-digested (top, left 2 panels; probe: *ARS313*/*ARS314*), *Bgl*II-digested (bottom, 2 panels; probe: *MET10/SMC2* (*ARS608*)) and *Eco*RV–digested (top, right 2 panels; probe: *tRNA*^Y^) DNA of the indicated strains and growth conditions. Replication pausing at the *ARS313*/*ARS314* locations is about three-fold, at the *ARS608* locations about four-fold, and at the *tRNA*^Y^ gene about seven-fold reduced in the CR-*rrm3* cells. Experiments were carried out two times. (**C**) Separation of circular from linear DNA structures by 2D gels (ethidium-bromide in both dimensions) of the indicated strains (WT, *rrm3*, *rad52*, *rrm3 rad52*) grown in medium containing 2% glucose or 0.05% glucose (CR). Structure A and B are indicated in the cartoon and appear only in CR-grown cells. (**D**) 2D gels of *Cla*I-digested DNA of the indicated strains and growth conditions. Probe: rDNA (*Stu*I-*Bgl*II). Pauses of replication forks at the natural RPS in rDNA are indicated by asterisks (*, **, ***; see also **A**).

Rrm3 is a DNA helicase involved in DNA replication^23,25–27^. We investigated therefore whether replication pausing/stalling is altered particularly at natural RPS (e.g., the inactive RFB, 5S rDNA genes, and silent rDNA replication origins (*ARS*)) (Fig. 2). To analyze rDNA replication intermediates by neutral–neutral DNA 2D agarose gel electrophoresis, we digested the DNA with the restriction enzyme *Cla*I which generates mainly an about 6 kb fragment from the 9.1 kb rDNA repeat unit (Fig. 2A)^27,79,81^.

As expected, and previously demonstrated, replication pausing/stalling at the inactive RFB, 5S rDNA genes and silent rDNA replication *ARS* was increased in the *rrm3* mutant compared to WT, both strains were grown in medium containing 2% glucose (Fig. 2A)^27^. Under conditions of CR, replication pausing/stalling at the inactive RFB and silent rDNA replication *ARS* was also increased in the *rrm3* mutant compared to WT, in contrast replication pausing/stalling did not increase at 5S rDNA genes in the CR-*rrm3* mutant cells (Fig. 2A). The rDNA replication pattern of WT cells grown in regular medium (2% glucose) and CR-medium were indistinguishable (P < 0.05; Fig. 2A).

Previously we demonstrated that replication pausing at the *tRNA*^A^ gene is reduced in CR-*rrm3* mutant cells^81^. Here we demonstrate that replication pausing/stalling at the silent replication origins *ARS313*, *ARS314* as well as *ARS608* and the *tRNA*^Y^ gene is also reduced in CR-*rrm3* mutant cells (Fig. 2B).

When we separated intact, undigested total genomic DNA in two dimensions in the presence of ethidium-bromide (in both dimensions^27^), and probed the membrane after the Southern-transfer with radioactive-labeled rDNA, we observed slower migrating DNA species (containing rDNA) particularly in CR cells (both WT and *rrm3* mutant cells; marked as structures A and B) (Fig. 2C). We also observed these DNA structures in the absence of the recombination protein Rad52 (*rad52* mutant background) (Fig. 2C). The amounts of ERCs (presence is dependent on Rad52) were similar in *rrm3* mutant cells grown in medium containing 2% glucose or 0.05% glucose (CR) (2% glucose: eightfold enrichment of ERCs in *rrm3* mutant over WT; CR: 8.2-fold enrichment of ERCs in *rrm3* mutant over WT; Fig. 2C). Replication patterns of rDNA in the *rad52* and *rrm3 rad52* strains display the same patterns observed in WT and *rrm3* strains, respectively (compare Fig. 2A,D).

In summary, these results suggest that in *rrm3* mutant cells, which were transferred from growth medium containing 2% glucose to CR-growth medium, replication pausing/stalling is in fact reduced likely genome-wide at numerous natural RPS (including tRNA and 5S rDNA genes as well as selective silent replication origins (*ARS*s)) to various extents. However, high-molecular weight structures in rDNA increase under CR-growth conditions in a Rad52-independent manner.

### Numbers of *rrm3* mutant cells stained with propidium iodide (PI) are low under normal growth conditions (2% glucose) and increased under caloric restriction (CR)

**Figure 3:**
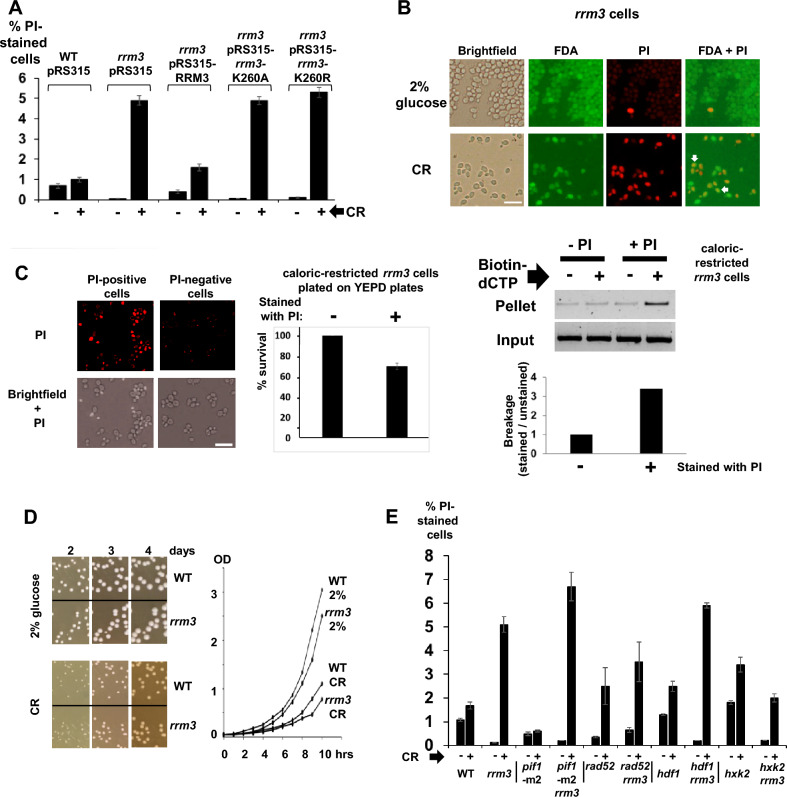
Numbers of propidium iodide (PI)-stained *rrm3* mutant cells (indicating dying or dead cells) growing in 2% glucose-medium are low and increased under caloric-restricted (CR) growth conditions. (**A**) The histogram displays the percentage of PI-stained cells. Standard errors are shown. Cells of the indicated strains were grown in medium containing 2% or 0.05% glucose to an early logarithmic phase (OD_600_ ~ 0.1). These are: WT, pRS315; *rrm3*, pRS315; *rrm3*, pRS315-*RRM3*; *rrm3*, pRS315-*rrm3* (K260A); *rrm3*, pRS315-*rrm3* (K260R). Representative microscopic images (PI-stained cells and the corresponding brightfield images) are displayed in Supplement Fig. 2. The two alleles *rrm3* (K260A) and *rrm3* (K260R) are properly expressed as previously demonstrated^27^. Experiments were carried out three times. (**B**) Microscopic images of *rrm3* mutant cells grown in medium containing 2% or 0.05% (CR) glucose. Cells were stained with fluorescein diacetate (FDA) and PI. In addition, the corresponding brightfield images are shown. Bar: 25 µm. (**C**) A population of cells containing PI-stained cells (CR *rrm3*) were sorted resulting in a fraction of cells enriched with stained cells (PI-positive). The other fraction contained non-stained cells (PI-negative). Brightfield images (superimposed with the PI-staining) are displayed below. Bar: 25 µm. Equal numbers of cells were plated on YEPD plates (2% glucose). Colonies were counted. The histogram shows percentage survival expressed as colonies on the PI-positive plate *versus* colonies on the PI-negative plate. Experiments were carried out three times. Standard errors are shown. DNA breakage in rDNA of cells stained or not stained with PI was determined by using the terminal deoxynucleotidyl transferase (TdT) assay combined with PCR as described in Fig. 1. Agarose gels display the PCR products for the rDNA fragment #1 (see also Supplement Fig. 1C). (−) and (+) biotin-dCTP refer to “no addition” or “addition” of biotin-dCTP to the TdT reaction. The histogram displays the quantification of the agarose gels on the left side as described in Fig. 1. (**D**) WT and *rrm3* mutant cells were plated on YEPD medium containing 2% or 0.05% (CR) glucose. Images display colonies after 2, 3, and 4 days of growth at 30 °C. Growth curves at 30 °C of WT and *rrm3* mutant cells grown in YEPD medium containing 2% or 0.05% (CR) glucose. (**E**) The histogram displays the percentage of PI-stained cells. Standard errors are shown. Cells of the indicated strains were grown in medium containing 2% or 0.05% glucose to an early logarithmic phase (OD_600_ ~ 0.1). These are WT, *rrm3*, *pif1-*m2, *pif1-*m2 *rrm3*, *rad52*, *rad52 rrm3*, *hdf1*, *hdf1 rrm3*, *hxk2*, *hxk2 rrm3*. Representative microscopic images (PI-stained cells and the brightfield images) are displayed in Supplement Fig. 3.

DNA breakage occurs in dying or dead cells because of the digestion of chromosomal DNA during the cell death process (e.g., apoptosis or necrosis)^82,83^. To determine the number of dying and dead cells, we added the dye propidium iodide (PI) to growing yeast cultures. PI can enter dying/dead cells and binds to DNA, because these cells have disrupted cell membranes^83^. Whereas fewer than 1% of the WT cells were PI-positive in YEPD medium containing 2% glucose, we discovered that *rrm3* mutant cells (2% glucose) had even ten times fewer PI-positive cells (0.07%; Fig. 3A; Supplement Fig. 2). The percentage of PI-positive *rrm3* mutant cells is greatly increased in CR-growth medium containing 0.05% glucose (i.e., from 0.07% (2% glucose) to 4.9% (CR); Fig. 3A; Supplement Fig. 2). This phenotype can be rescued by expression of the functional *RRM3* gene, whereas expression of mutant versions of *rrm3* (K260A, K260R), which affect the binding of ATP to Rrm3 (K260A) and the hydrolysis of ATP (K260R), resemble the same effect as a complete lack of Rrm3 (Fig. 3A; Supplement Fig. 2)^27^. The mutant *rrm3* versions (K260A, K260R) are expressed at WT levels as previously demonstrated^27^.

Whether PI-stained cells are indeed dead/dying cells, we applied two approaches. We were co-staining *rrm3* mutant cells grown in medium containing 2% or 0.05% (CR) glucose with fluorescein diacetate (FDA) and PI (Fig. 3B)^84^. Living cells are stained in green (FDA), whereas dead cells (or undergoing cell death) display a red/orange color (PI). We noticed that some of the CR-*rrm3* cells were stained both in green and red/orange (indicated with arrows; Fig. 3B). We propose that these cells are in a transition-state to cell death. In the second approach, we enriched PI-stained cells by cell sorting (Fig. 3C). Whereas one fraction contained cells (here: CR-*rrm3* mutant cells) with 25–50% PI-positive cells, the other fraction contained cells, which were not stained with PI (PI-negative cells; Fig. 3C). Equal numbers of cells of these two fractions were spread on YEPD plates. About 70% of the fraction containing PI-stained cells formed viable colonies (Fig. 3C). An aliquot of these fractions (stained or not stained with PI) was also used to purify intact genomic DNA and was used to determine DNA breakage by PCR as shown in Fig. 1. Broken DNA (rDNA) is mainly detected in the fraction with the PI-stained cells (Fig. 3C). Regarding the general growth behavior, colonies of WT and *rrm3* mutant cells on plates containing medium with 2% or 0.05% glucose were of similar size, respectively (Fig. 3D). CR cells (both WT and *rrm3* mutant background) had a 5–10% slower growth curve (Fig. 3D).

We were analyzing loss-of-function mutations of several genes (i.e., deletion mutations) to address whether they affect (e.g., promote, suppress) the increase of the rates of PI-stained cells, when *rrm3* deletion mutant cells are switched from normal glucose levels (i.e., 2% glucose) to CR-restricted growth conditions (0.05% glucose). For example, the nuclear proteins Pif1, Rad52 and Hdf1, which are all involved in DNA repair/maintenance mechanisms, may promote/affect the formation of potential toxic DNA intermediates under CR growth conditions.

We tested mutants lacking the homolog of Rrm3, the DNA helicase Pif1^85,86^. Since Pif1 is present in both the mitochondria and the nucleus, we used mutants only lacking the nuclear form of Pif1 (*pif1*-m2)^85^. The *pif1*-m2 mutant strain does not have a mitochondrial defect (i.e., petite growth phenotype). The double mutant strain *rrm3 pif1*-m2 shows the same trend as the single mutant strain *rrm3.* We observed that the number of PI-stained cells increases in *rrm3 pif1*-m2 from 0.2% (in 2% glucose) to 6.7% (in 0.05% glucose), and in the *rrm3* mutant from 0.13% (in 2% glucose) to 5.1% (in 0.05% glucose) (Fig. 3E; Supplement Fig. 3), the increase in the double mutant is about 30% larger. The number of PI-stained cells remains low in the *pif1*-m2 single mutant in both under CR (0.6%) and non-CR (0.48%) growth conditions and are lower compared to WT (Fig. 3E; Supplement Fig. 3).

We investigated whether lack of the repair genes Rad52 and Hdf1, which are involved in homologous and non-homologous DNA repair, have an influence on the percentage of PI-stained cells in *rrm3* mutant cells^87–89^. Neither lack of Rad52 nor Hdf1 has any influence on the increase in the number of PI-positive cells, when *rrm3* mutant cells are transferred from growth medium containing 2% glucose to CR-growth medium (Fig. 3E; Supplement Fig. 3). However, an increase in the number of PI-stained cells can be observed in the single mutants (*rad52*, *hdf1*), when these cells are transferred from medium containing 2% glucose to CR-growth medium (Fig. 3E; Supplement Fig. 3).

Hxk2 encodes one of three hexokinases involved in converting glucose into glucose-6-phosphate, thus introducing glucose into the glycolysis pathway^90^. Absence of the non-essential Hxk2 protein has similar effects on cells compared to CR-growth conditions in some genetic backgrounds^1,91,92^. We determined the frequency of the appearance of PI-stained cells of *hxk2* and *rrm3 hxk2* mutant strains grown in medium containing 2% glucose and CR (Fig. 3E). Interestingly, whereas under CR-growth conditions in all other combination (WT *versus rrm3*, *pif1*-m2 *versus pif1*-m2 *rrm3*, *rad52 versus rad52 rrm3*, *hdf1 versus hdf1 rrm3*) the number of PI-stained cells increases, there is a decrease in these numbers, when *hxk2* is compared to *rrm3 hxk2* suggesting the increase in cell death in the CR-*rrm3* cells is partially independent of the glucose concentration (Fig. 3E; Supplement Fig. 3).

In summary, (i) though the DNA breakage rates (Fig. 1) and appearance of PI-stained cells (Fig. 3) follow similar trends to some extent, there are differences. Whereas the increase in DNA breakage rates of *rrm3* cells after transferring from medium containing 2% glucose to CR medium does not reach WT-levels for most of the tested DNA fragments (except for rDNA), numbers of PI-stained (and presumably dying or dead) cells are fivefold higher in CR-*rrm3* cells compared to WT cells (Fig. 1). (ii) The absence of Pif1, Rad52 or Hdf1 has no influence on the increase in the number of PI-stained cells of *rrm3* mutant cells (comparison of WT with the *rrm3* mutant, and single mutants (*pif1*-m2, *rad52*, *hdf1*) with *rrm3* double mutants) under CR-growth conditions. (iii) In contrast, when *hxk2* and *rrm3 hxk2* mutant strains are compared under CR-growth conditions, the number of PI-stained cells decrease in the *rrm3 hxk2* mutant strain.

### The checkpoint protein Rad53 is less (or not at all) phosphorylated in the *rrm3* single and other *rrm3-*combination mutants under CR

**Figure 4:**
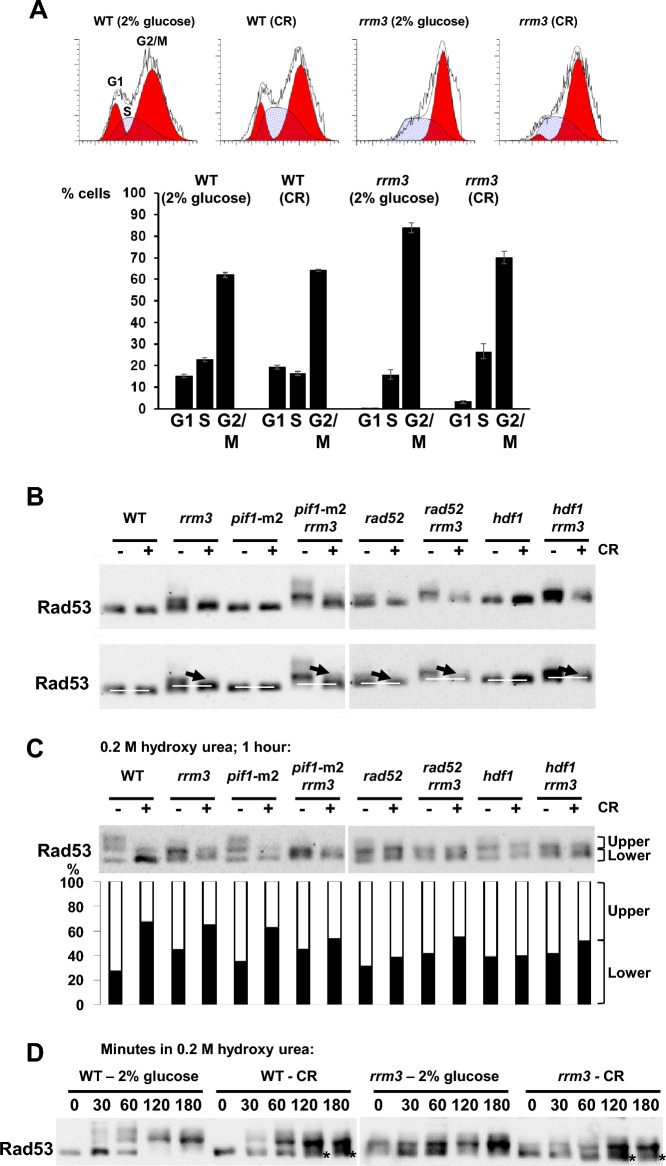
Increased numbers of cells in S-phase when *rrm3* mutant cells are grown under caloric-restricted (CR) growth conditions and lower levels of Rad53-phosphorylation in CR-*rrm3* cells and several CR-*rrm3* double mutations. (**A**) Cell cycle distribution of WT and *rrm3* mutant cells, grown in medium containing 2% or 0.05% (CR) glucose, determined by FACS analysis. The distribution of cells in G1, S and G2/M were modelled according to the ModFit LT program. Percentages of cells with 1N, 2N and intermediate DNA content are displayed as a histogram. Standard errors are shown. (**B**) Western-blots probed for Rad53p of the indicated strains and grown in YEPD containing 2% (CR: −) or 0.05% (CR: +) glucose. (**C**) Western-blots probed for Rad53p of the indicated strains and grown in YEPD containing 2% (CR: −) or 0.05% (CR: +) glucose in the presence of 0.2 M hydroxy urea (HU) for one hour. Percentage-distributions are displayed below. (**D**) Time course experiment with WT and *rrm3* mutant cells grown in medium containing 2% or 0.05% (CR) glucose and containing 0.2 M HU. Samples were taken at the indicated times and probed for Rad53p by western-blotting. Asterisks indicate Rad53 protein species which were still non-phosphorylated under CR-growth conditions. Experiments in (**A**–**D**) were carried out three times. Images of the western-blots in full size are displayed in supplementary figures.

The *rrm3* mutant has a delay in the cell cycle when cells pass through the G2/M phases^23,25^. We determined by FACS analysis whether the cell cycle profiles of WT and *rrm3* mutant cells alter when cells are switched to CR-growth conditions (Fig. 4A, FACS). There are 22.8% of the WT cells in S-phase in medium containing 2% glucose, and 16.5% S-phase cells in CR-medium (1.4-fold decrease). There are 62.0% of the WT-cells in G2/M-phase in medium containing 2% glucose, and 64.2% of the cells in G2/M-phase in CR-medium. There are 15.2% of the WT-cells in G1-phase in medium containing 2% glucose, and 19.3% of the cells in G1-phase in CR-medium, which is a 1.3-fold increase. In contrast, whereas 83.9% of *rrm3* cells grown in medium containing 2% glucose reside or pass slowly through the G2/M phases, the number of cells in these phases drop to 70.1% in CR-growth medium. The number of *rrm3* cells in S-phase increases from 15.8% to 26.6% (1.7-fold increase), and in G1-phase from 0.24% to 3.2% (13.3-fold increase) when cells are switched to CR-growth medium.

Since DNA damage occurs in *rrm3* mutant cells which activates the intra S-phase checkpoint^26,58,59^, we determined the status of the Rad53 protein phosphorylation in WT, various single mutant strains (i.e., *rrm3*, *pif1*-m2, *rad52*, *hdf1*) and *rrm3* double mutant strains (i.e., with *pif1*-m2, *rad52*, *hdf1*) in growth medium containing 2% or 0.05% (CR) glucose (Fig. 4B). As previously demonstrated, as an indication for DNA damage Rad53 is phosphorylated in *rrm3* cells, which were grown in medium containing 2% glucose (Fig. 4B)^26,58,59^. However, Rad53 is phosphorylated at largely reduced levels in CR-*rrm3* cells (Fig. 4B). Similar phenotypes appear also in the *pif1*-m2 *rrm3*, *rad52 rrm3* and *hdf1 rrm3* double mutants, i. e. intense phosphorylation of Rad53 in cells grown in 2% glucose and reduced/missing phosphorylation in the corresponding CR-cells (Fig. 4B). Rad53 protein exhibits limited phosphorylation in *rad52* cells grown in medium containing 2% glucose which is markedly reduced in CR-*rad52* cells (Fig. 4B). No phosphorylation of Rad53 occurs in WT, *pif1-*m2 and *hdf1* mutant cells (grown in medium containing 2% glucose or grown under CR-conditions) (Fig. 4B).

Since no phosphorylation of Rad53 is observed in CR-*rrm3* cells, we investigated whether the checkpoint can still be activated. We determined the status of Rad53 phosphorylation in cells experiencing induced DNA damage and growing at normal glucose levels (i.e., 2%) or under CR (i.e., 0.05%). We treated WT, various single mutants (i.e., *rrm3*, *pif1*-m2, *rad52*, *hdf1*) and *rrm3* double mutants (i.e., with *pif1*-m2, *rad52*, *hdf1*) with hydroxy-urea (HU; 0.2 M, final concentration) for 1 h (Fig. 4C). Rad53 protein in WT and *pif1-*m2 cells grown in 2% glucose and in the presence of HU is highly phosphorylated; in contrast, levels of Rad53 protein phosphorylation are markedly reduced in the corresponding CR-cells (Fig. 4C). Although in the single mutants *rrm3*, *rad52*, *hdf1*, and the *rrm3* double mutants (i.e., with *pif1*-m2, *rad52*, *hdf1*) Rad53 protein is phosphorylated in cells grown both in 2% glucose and in CR-medium, the non-phosphorylated form is more abundant under CR-growth conditions (except in the case of *hdf1*) (Fig. 4C, histogram). Further, in a time course experiment we determined that in WT cells grown in medium containing 2% glucose and HU, Rad53 is activated (by phosphorylation) faster compared to WT cells grown under CR growth conditions or in *rrm3* mutant cells grown in HU-medium containing 2% glucose or 0.05% (CR) (Fig. 4D; asterisks indicate remaining non-phosphorylated form in CR-cells).

In summary, although Rad53 does not appear to be phosphorylated in CR-*rrm3* cells in unchallenged conditions (in contrast to *rrm3* cells grown in medium containing 2% glucose), CR-*rrm3* cells under induced DNA damage conditions does result into phosphorylation of Rad53, thus the damage response mechanisms in the CR-*rrm3* mutant cells appear to be functional.

### The Sir2/Sir3/Sir4 complex is needed for the increase of the number of PI-stained cells of CR-*rrm3* mutant cells

**Figure 5:**
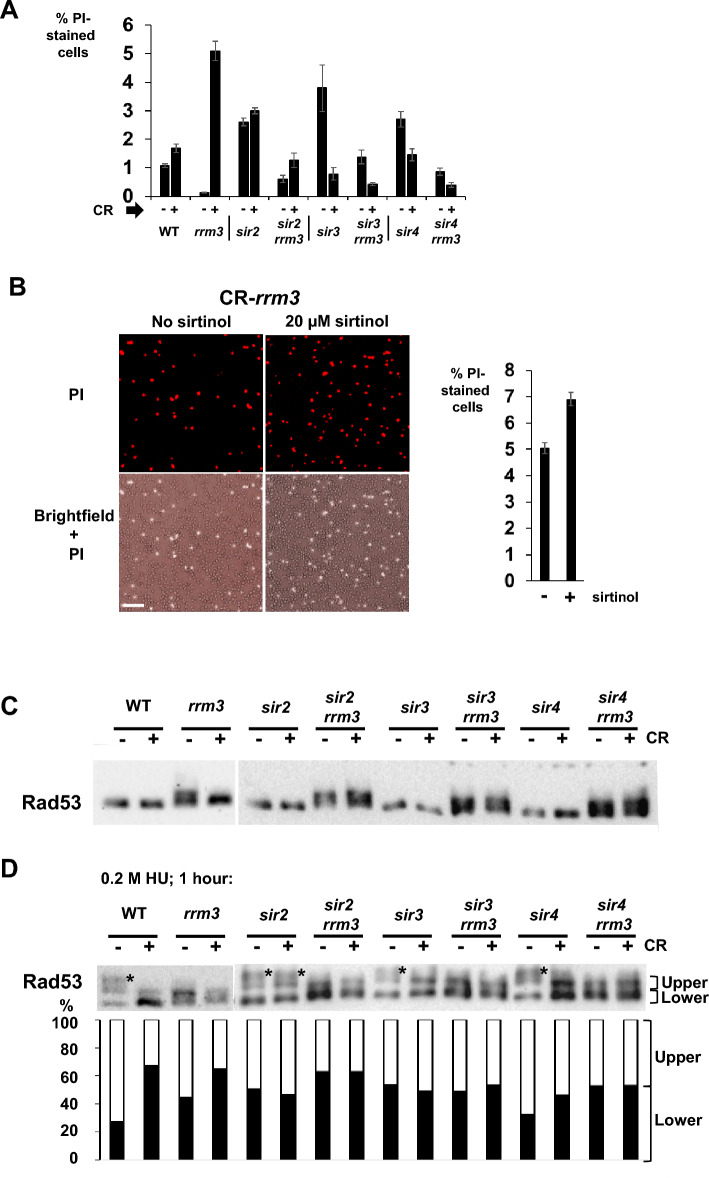
The Sir2/3/4 proteins are needed for the increase in cell death rates in the *rrm3* mutant after switch to CR-restricted growth conditions. (**A**) The histogram displays percentage of propidium iodide (PI)-stained cells. Standard errors are shown. Cells of the indicated strains were grown in medium containing 2% or 0.05% glucose to an early logarithmic phase (OD_600_ ~ 0.1). These are WT, *rrm3*, *sir2*, *sir2 rrm3*, *sir3*, *sir3 rrm3*, *sir4*, *sir4 rrm3*. Representative microscopic images (PI-stained cells and the brightfield images) are displayed in Supplement Fig. 4. (**B**) *rrm3* mutant cells were grown under CR conditions in the absence or presence of the Sir2-inhibitor sirtinol (20 µM final concentration). Microscopic images display PI-stained cells (orange) and the corresponding brightfield images (PI-staining superimposed). Bar: 50 µm. The histogram displays the percentage of PI-stained cells. Experiments were carried out three times. Standard errors are shown. (**C**) Western-blots probed for Rad53p of the indicated strains and grown in YEPD containing 2% (CR: −) or 0.05% (CR: +) glucose. The image on the left side (WT, *rrm3*; 2% and 0.05% glucose) is taken from Fig. 4B for comparison. Experiments were carried out two times. (**D**) Western-blots probed for Rad53p of the indicated strains and grown in YEPD containing 2% (CR: −) or 0.05% (CR: +) glucose in the presence of 0.2 M hydroxy urea (HU) for one hour. Asterisks indicate the fully phosphorylated Rad53 protein. The image on the left side (WT, *rrm3*; 2% and 0.05% glucose) is taken from Fig. 4C for comparison. Percentage-distributions are displayed below. Experiments were carried out two times. Images of the western-blots in full size (**C**,**D**) are displayed in supplementary figures.

Given there is a relationship between glucose restriction, repressive chromatin, and presence/activity of Rad53^64^, we investigated whether absence of any member of the Sir-complex affects the increase in the number of PI-stained cells when *rrm3* cells are transferred from normal growth medium (2% glucose) to CR-growth medium (0.05%). In all single deletion mutants of the Sir-complex (*sir2*, *sir3* and *sir4*) the percentage of PI-stained cells is elevated compared to WT in medium containing 2% glucose (WT: 1.1%; *sir2*: 2.6%; *sir3*: 3.8%; *sir4*: 2.7%; Fig. 5A; Supplement Fig. 4). Under CR growth conditions in both single (*sir2*, *sir3* and *sir4*) and double mutants (*sir2 rrm3*, *sir3 rrm3* and *sir4 rrm3*), the percentage of PI-stained cells is either only minimally increased (*sir2*: 2.6% (2% glucose) and 3% (CR); *sir2 rrm3*: 0.61% (2% glucose) and 1.3% (CR)) or even decreased (*sir3*: 3.8% (2% glucose) and 0.78% (CR); *sir4*: 2.7% (2% glucose) and 1.5% (CR); *sir3 rrm3*: 1.4% (2% glucose) and 0.43% (CR); *sir4 rrm3*: 0.86% (2% glucose) and 0.39% (CR)) (Fig. 5A; Supplement Fig. 4).

The Sir2-inhibitor sirtinol reduces the enzymatic activity of the Sir complex (Sir2/3/4)^93^. Interestingly, culturing of *rrm3* mutant cells in CR-medium in the presence of sirtinol causes an increase in the number of PI-stained cells (Fig. 5B).

In summary, absence of the Sir2/Sir3/Sir4 complex decreases the numbers of PI-stained cells in *rrm3* mutant strains under CR-growth conditions (Fig. 5A; Supplement Fig. 4) though the Sir2 protein may have a separate/additional role in this process (e.g., Sir2, but not Sir3/4, is present in the nucleolus).

### The checkpoint protein Rad53 is phosphorylated at about the same levels in *rrm3 sir2*, *rrm3 sir3*, or *rrm3 sir4* mutants grown in medium containing 2% glucose or in CR-medium

We determined the status of the Rad53 protein phosphorylation in both single (*sir2*, *sir3* and *sir4*) and double mutants (*sir2 rrm3*, *sir3 rrm3* and *sir4 rrm3*) in growth medium containing 2% or 0.05% (CR) glucose (Fig. 5C). Rad53p is phosphorylated equally in the double mutant *rrm3* (with *sir2*, *sir3* and *sir4*) cells grown in medium containing 2% or 0.05% glucose (Fig. 5C). Also, here we investigated the status of Rad53 phosphorylation in cells experiencing induced DNA damage and growing at normal glucose levels (i.e., 2%) or under CR (i.e., 0.05%). We treated WT, the single mutants (*rrm3*, *sir2*, *sir3* and *sir4*) and *rrm3* double mutants (*sir2 rrm3*, *sir3 rrm3* and *sir4 rrm3*) with hydroxy-urea (HU; 0.2 M, final concentration) for 1 h (Fig. 5D). Whereas in WT and *rrm3* cells grown in 2% glucose Rad53 protein is highly phosphorylated and at a reduced level in the corresponding CR-cells, Rad53 is phosphorylated at similarly high levels in all the sir-mutants in cells grown both in 2% glucose and in CR-medium (Fig. 5D, histogram).

### Mitochondrial oxidative stress contributes to the increase in PI-stained cells of the *rrm3* mutant under CR growth conditions

Cell death including apoptosis may involve and be initiated by the opening of the mitochondrial structure (e.g., opening of the MTP (mitochondrial permeability transition pore), rupture of the outer mitochondrial membrane with release of cytochrome c) which may lead to an increase in oxidative stress^94,95^. Using fluorescence microscopy, we visualized which cells exhibit excessive oxidative stress and undergo cell death. Cells with excessive numbers of reactive oxygen species (ROS) were visualized by using the Singlet Oxygen Sensor Green (SOSG, Molecular Probes) reagent, and cells potentially undergoing cell death were detected with PI as described above (Supplement Fig. 5)^96^. Whereas almost all PI-stained cells had clear overlapping SOSG signals in WT and *rrm3* mutant cells (both with medium containing 2% glucose and CR-medium), there were additional cells with excessive ROS (compared to the number of PI-positive cells) under normal growth conditions (i.e., 2% glucose) both in WT and *rrm3* mutant cells, which however did not appear (yet) to undergo cell death (PI-positive) (WT: ~ 50% increase; *rrm3*: ~ 100% increase)(SOSG-positive/PI-negative cells labeled with asterisk; Supplement Fig. 5A). In contrast, almost all the WT and *rrm3* mutant cells grown in CR-medium, which showed excessive oxidative stress (i.e., green staining by SOSG) were also PI-positive (i.e., likely undergoing cell death), there were almost no SOSG-positive cells, which were not PI-positive.

Calorie restriction increases respiration activity^74^. We were co-staining cells with SOSG and MitoTracker Red CMXRos, which is a red-fluorescent dye that stains mitochondria in live cells and which reflects the intensity of membrane potential (i.e., respiration activity; Supplement Fig. 5B). In general, cells grown under CR conditions (both WT and *rrm3* mutant cells) exhibit stronger staining with MitoTracker Red CMXRos reflecting higher respiration activity.

To investigate whether an increase in mitochondrial oxidative stress is a prerequisite for the increase in the numbers of PI-positive cells in the CR-grown *rrm3* mutant cells, we treated CR-*rrm3* mutant cells with the compound Mito-TEMPO (SigmaAldrich, St. Louis, MO) which reduces oxidative stress in mitochondria^97^. Treating CR-grown *rrm3* mutant cells in the presence of 160 µM Mito-TEMPO reduces the number of cells with PI-positive signals to about 50% of untreated CR-*rrm3* mutant cells (Supplement Fig. 5C).

In summary, minimizing mitochondrial oxidative stress decreases the cell death rates in CR-*rrm3* mutant cells.

## Discussion

Growth conditions of calorie restriction (CR) usually induce lifespan extension, improvement of tissue quality, and an increase in genome stability^5^. We discovered that the chromosomal DNA breakage as well as survival rates of the *rrm3* DNA helicase mutant are impacted by CR-growth conditions. By using the terminal deoxynucleotidyl transferase (TdT) assay we observed that despite the presence of about 1400 chromosomal replication pause sites (RPS) DNA breakage rates of *rrm3* mutant cells were lower compared to WT in normal growth medium (i.e. 2% glucose) (Fig. 1). However, after transferring the cells to CR-growth conditions (i.e. 0.05% glucose) DNA breakage rates increased in the *rrm3* mutant. Except for rDNA, where DNA breakage rates were higher in the CR-*rrm3* mutant compared to WT, DNA breakage rates in the CR-*rrm3* mutant did not reach WT levels at numerous other (non-rDNA) genomic loci (Fig. 1). The numbers of PI-stained cells, which reflect cell death rates, mirrored the DNA breakage rates to some degree, however there were a few differences (Fig. 3). Though the cell death in WT and *rrm3* mutant cells may have different causes, cell death rates are lower in the *rrm3* mutant compared to WT when grown in medium containing 2% glucose. One possibility to interpret this is that *rrm3* mutant cells (in 2% glucose medium) have numerous replication defects which activate the S-phase checkpoint^25,26,58,59^. These conditions lead to a delay in the cell cycle (around G_2_/M phases) to allow repair of any defects, thus cell death rates are reduced. However, under CR-growth conditions cell death rates increase in the *rrm3* mutant to fivefold higher levels (compared to CR-WT cells), which contrasts with the changes in DNA breakage rates (for most of the tested genomic loci) in the CR-*rrm3* mutant. Though it is unknown what the primary cause of the increase in cell death rates in the *rrm3* mutant is after transfer of the cells from 2% glucose medium to CR-growth medium, the cell death events (in the *rrm3* mutant) might be the consequence of single deleterious events in individual cells. For example, loss of a single telomere may lead to the loss of the affected chromosome and consequently to cell death^98^. In individual cells, different chromosome parts might be prone to severe damage/loss, which may lead to cell-death as an overlapping result in all affected cells. DNA breakage events may occur in individual cells at different genomic locations, this might be a possible explanation why the DNA breakage rates are lower at individual sites compared to the overall cell death rates. rDNA might be an exception because it is a repeated sequence. Further, there are differences in the cell cycle-distribution in *rrm3* cells grown in 2% glucose *versus* CR-growth conditions (Fig. 4), which may also contribute to a difference in the breakage rates. In addition, the measured DNA breakage events might result from different events. Breakage events may occur during deleterious replication/recombination processes in chromosomal DNA, but may also be the result of cells undergoing cell death as a consequence of a deleterious chromosomal damage event that cannot be repaired (i.e., degradation of chromosomal DNA). There is however an indication that the majority of the detected breakage events originate from the dead cells (see sorting experiment in Fig. 3C). Determining the locations of DNA breaks on potentially single-cell level is a direction to address where DNA breaks initially before cells may enter the cell death pathway^99–101^.

Interestingly, the Rad53 checkpoint protein is phosphorylated at largely reduced levels in CR-*rrm3* mutant cells (Fig. 4). The Rad53 checkpoint protein normally ensures that cells (transiently) arrest, when DNA is damaged (e.g., broken DNA as a possible consequence of stalled replication forks) until the damage is repaired^102,103^. We demonstrate that replication fork pausing/stalling at tRNA and 5S rDNA genes as well as at selective silent replication origins is in fact reduced/missing in the CR-*rrm3* cells compared to *rrm3* cells propagated in medium containing 2% glucose (Fig. 2). There are about 270 tRNA genes, 100–150 5S rDNA genes and 50–100 (or more) silent replication origins in the haploid yeast genome. Conditions of CR lead to a down-regulation of protein synthesis activities which include translation regulation, ribosome synthesis and assembly as well as mRNA export from the nucleus^104–106^. Recently we also found less binding of Tfc1 (part of the pre-initiation complex) to 5S rDNA and tRNA genes in CR-cells, which may explain the largely reduced replication pausing at 5S rDNA and tRNA genes in the CR-*rrm3* mutant cells^81^. Alternatively, the reduced replication pausing/stalling phenotype may also be the result of the higher expression of Rrm3p-like helicases in CR-*rrm3* mutant cells.

The reduction in pausing/stalling at tRNA and 5S rDNA genes as well as at selective silent replication origins would fit to the data with the reduced phosphorylation rates of Rad53 in CR-*rrm3* cells assuming that paused forks may also stall and collapse leading to Rad53 activation by phosphorylation. Since about 5% of the CR-*rrm3* cells are undergoing cell death (stained with PI; Fig. 3), we speculate that the non-phosphorylated form of Rad53 and the reduced pausing/stalling at the RPS originates (likely mainly) from the 95% remaining living *rrm3* cells. Western-blotting and DNA 2D gels are not sensitive enough to detect protein-modifications and DNA structures, respectively, only occurring in the 5% cell population which represent the dying cell population. However, Rad53 protein can be activated by induced DNA damage in CR-growth medium (by exposing to HU treatment; Fig. 4), thus if DNA damage (such as broken replication forks or long single-stranded DNA tracts) is present, Rad53 can be activated by phosphorylation. We propose therefore that Rad53 protein residing in the 95% cell population of the living CR-*rrm3* cells is (almost) not activated under non-challenging conditions, thus DNA breakage may occur only at an extremely low frequency (e.g., in a few single cells).

Another possibility to explain the elevated levels of non-phosphorylated Rad53 in the CR-*rrm3* mutant is that more cells of this mutant are in S-phase as demonstrated by FACS analysis (Fig. 4A). Schleker et al*.* demonstrated that Rad53 exhibits a small phosphorylation-dependent electrophoretic mobility shift in G_2_, M and G_1_ phases of the cell cycle that is missing in S-phase and that occurs independently of nuclear DNA damage^107^. The G_2_, M and G_1_ phases-dependent phosphorylation of Rad53 appears to be accomplished by the Cdc5 and Cdc28 protein kinases, but the loss of phosphorylation during S-phase does not depend on the protein phosphatases Ptc2 and Ptc3, which are normally involved in checkpoint adaptation^107^. However, under CR-growth conditions other protein phosphatase may dephosphorylate Rad53 efficiently such as Irc21, which acts together with other protein phosphatases including PP2A^108^. A proposed reason for the fluctuation in Rad53 phosphorylation is that the checkpoint response is attenuated in S-phase to allow for naturally occurring single-strand DNA at the replication forks^107,109,110^. In unchallenged cells temporarily activation of Rad53 at the beginning of S-phase has also the purpose of raising the amount of the dNTP pool which is needed during a faithful DNA replication process^111^.

**Figure 6:**
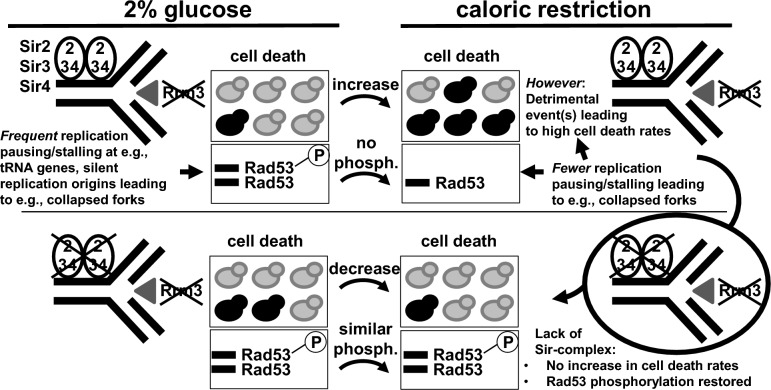
The Sir protein complex is needed for the increase in cell death rates in the *rrm3* mutant after switch to CR-restricted growth conditions. In CR *rrm3* mutant cells cell death rates are increased compared to cells grown in regular growth medium (2% glucose), this increase in cell death rates is prevented in the absence of the Sir complex (Sir2/Sir3/Sir4). The Sir-complex is present at telomeres and the silent mating type loci *HML* and *HMR*. Whereas the Rad53 checkpoint is activated (i.e., phosphorylated) in *rrm3* cells grown in regular growth medium (2% glucose), Rad53 is not activated in CR-*rrm3* cells. Rad53 phosphorylation is restored in CR-*rrm3 sir2*/*3*/*4* cells.

What could be the reason why *rrm3* mutant cells undergo increased cell death when transferred from 2% glucose medium to CR-medium (Fig. 6)? We demonstrate here that the increase in cell death rates in the *rrm3* mutant after shifting cells from 2% glucose medium to CR-growth conditions is dependent on the Sir-complex (Sir2/3/4), but appears to be independent of Pif1, Rad52 or Hdf1 (Figs. 3 and 5). Whereas there is still a small increase in PI-positive cells in the *sir2 rrm3* mutant after transferring cells from 2% glucose-medium to CR-growth medium, there is clearly a reduction in PI-positive cells when *sir3 rrm3* or *sir4 rrm3* mutant cells are transferred to CR-growth medium (Fig. 5). Whereas Sir2 alone can be found localized to the rDNA locus, the Sir complex consisting of the Sir2/Sir3/Sir4 proteins binds to telomeres and sub-telomeric regions as well as to the silent mating type loci *HML* and *HMR* (and surrounding area)^65^. Besides the well-known function of the Sir complex in silencing of genes at telomeres, the silent mating type loci or the rDNA locus, the Sir proteins also affect genome stability. For example, the Sir complex (Sir3 and Sir4) may promote non-homologous-end-joining (NHEJ), but there are also reports that Sir4 inhibits NHEJ, which may induce telomere end fusions^112–114^. Previously we described that in the absence of Rrm3 replication forks pause (or potentially stall) within telomeric DNA and the silent mating type loci *HML* and *HMR*, and that this pausing/stalling is still occurring in the absence of the Sir-complex (Sir2/Sir3/Sir4)^26^. Interestingly, we found that in the CR-*sir3 rrm3* or *sir4 rrm3* double mutant cells Rad53 protein is activated (phosphorylated; two bands visible) in contrast to the CR-*rrm3* single mutant in which Rad53 does not appear to be activated (not phosphorylated; only one band visible on the western blot) (Fig. 5). The Sir-complex has known structural and enzymatic roles at telomeres and the silent mating type sites^65^. Under CR growth conditions, Rrm3 may have an important (essential) function as a helicase when the replication fork passes through these Sir-protein-complex covered regions (or at other Sir-dependent regions). In this scenario when Rrm3 is absent, telomeres which are just being replicated may be prone to higher breakage rates because of unfinished replication leading possibly to a chromosome without a telomere. In addition, CR-*rrm3* mutant cells treated with the Sir2 inhibitor sirtinol have an increased cell death rate (Fig. 5B) which suggests that an enzymatic activity (e.g., deacetylation) linked to the Sir complex may also be involved in causing the high cell death rates of the CR-*rrm3 sir2* cells. Numerous mammalian and yeast studies demonstrate also that specific histone modifications (e.g., acetylation, methylation, ubiquitination) are important features of the cell death program^115^. For example, the gamma-H2AX phosphorylation-modification, which occurs during the formation of dsDNA breaks, is also present during apoptosis as well as phosphorylation of histone H2B at serine-14 is associated with chromatin condensation and DNA fragmentation (references in^115^). Future studies will address why Rad53 is phosphorylated in the CR-*sir3 rrm3* or *sir4 rrm3* double mutant cells but not in the CR-*rrm3* single mutant cells as well as we will address the possible role of chromatin modifications in the increased cell death rates in the CR-*rrm3* cells.

Whereas *rrm3* mutant cells grown in 2% glucose medium pass with a delay through the G_2_/M phases, the number of cells in S-phase increases when *rrm3* mutant cells are transferred to CR-growth conditions (Fig. 4). The cell-cycle arrest/delay during the DNA damage checkpoint in the G_2_/M phases is mainly regulated by the chaperone protein Pds1 (securin in mammals) and its binding partner, the cohesin protease Esp1 (separase)^116,117^. Pds1 binds Esp1 in undamaged cells and inhibits its protease activity^118^. When cells transit from metaphase to anaphase, Pds1 is ubiquitinated by the E3 ubiquitin ligase APC-Cdc20^119^. It is then degraded by the proteasome. Degradation of Pds1 releases Esp1 to cleave cohesin rings. This process allows sister chromatids to segregate. When DNA is damaged, Pds1 is phosphorylated by Chk1, blocking its interaction with APC-Cdc20^116^. Cdc20 is phosphorylated by Rad53, which prevents APC-Cdc20 from ubiquitinating Pds1^120^. Together, both Pds1 and Cdc20 phosphorylation inhibit mitosis progression by keeping Esp1 inactive. Deletion of Pds1 reduces the duration of G_2_/M arrest by 60% while arrest is completely abrogated in *pds1* *rad53* mutants^121,122^. It will be interesting to test whether Cdc20 is not phosphorylated by Rad53 in CR-*rrm3* mutant cells thereby releasing cells into M-phase before DNA damage is repaired.

Numerous proteins interact with Rrm3 on a genetic and/or functional level to affect telomeric or replicative processes^40,58,59,123–125^. Examples are the fork protection complex Tof1/Csm3 and Mrc1, proteins that are present at telomeres including the Mre11/Rad50/Xrs2 complex, or the cullin Rtt101. Viability of *rtt101* mutants depends on Rrm3^123^. Further, the increased cell death rates in the CR-*rrm3* mutant are also induced by mitochondrial reactive oxygen species (ROS) since treatment with the drug Mito-TEMPO, which reduces mitochondrial oxidative stress, lowers the cell death rates in the CR-*rrm3* mutant cells (Supplement Fig. 5C). It remains to be established how these genetic and metabolic factors interact to elucidate why *rrm3* mutant cells in CR growth medium experience higher cell death rates compared to *rrm3* mutant cells grown in medium 2% glucose. Though the genomic-instability mutant *rrm3* behaves worse under CR-conditions, dietary restriction for example in progeroid DNA repair-deficient mice (DNA repair-deficient Ercc1^∆/-^ mice) dramatically extend health span and tripled life span^126^. Thus, future studies are needed to explore how nuclear genomic instability, which is a prominent feature associated with cancer, affects cell survival under caloric/dietary restriction.

## Methods

### Experimental model and subject detail

We used haploid WT and mutant *S. cerevisiae* strains (*rrm3*, *sir2*, *sir3*, *sir4*, *pif1-*m2, *rad52*, *hdf1*, *hxk2*, *rrm3 sir2*, *rrm3 sir3*, *rrm3 sir4*, *rrm3 pif1-*m2, *rrm3 rad52*, *rrm3 hdf1*, *rrm3 hxk2*) (a list of used yeast strains is provided in the Supplemental Experimental Procedures as Table S1)^23,26,27,85^. *HXK2* was knocked out by *LEU2* as described previously^127^.

### Method details

#### Yeast methods, cell growth, incubation with drugs

Yeast strains were thawed from frozen stocks, streaked out on YEPD (yeast extract, pepton, dextrose) or selection plates containing 2% glucose and grown at 30 °C 2–3 days until small colonies were visible. Colonies were suspended in YEPD growth medium containing 2% glucose and grown for 6 h. Optical density (OD at 600 nm) was determined, cells were grown in YEPD growth medium containing 2% (regular level) or 0.05% (caloric restriction, CR) glucose to an early-logarithmic growth phase (about 1 × 10^7^ cells/ml). This means, usually cells were diluted to an OD_600_ of 0.001, grown to an OD_600_ of 0.1 (12–15 h) and then resuspended in fresh growth medium (containing 2% or 0.05% glucose) for about two hours to avoid growth limitations. Strains harboring the plasmids pRS315 (empty plasmid (*LEU2*)) or pIA20 (pRS315-*RRM3*, pRS315-*rrm3* (K260A), pRS315-*rrm3* (K260R)) were maintained in synthetic growth medium lacking the amino acid leucine^27^. Sirtinol (Sigma-Aldrich, St. Louis, MO) was dissolved in DMSO at a concentration of 5 mM and used in the experiment at a final concentration of 20 µM. Mito-TEMPO (Sigma-Aldrich, St. Louis, MO) was dissolved in DMSO at a concentration of 20 mM and used in the experiment at a final concentration of 160 µM.

### Determination of the DNA breakage rates using the terminal deoxynucleotidyl transferase (TdT) assay combined with PCR

The TdT assay is used to map the locations of double-strand DNA (dsDNA) breaks on a genome-wide scale by end-labeling of the DNA ends^77^. We purified highly intact whole genomic DNA and performed the TdT assay reaction using biotinylated dCTP. DNA was digested with a restriction enzyme (i.e., *Bgl*II) and the DNA fragments with the extended biotinylated tags (indicating DNA breakage) were recovered using avidin-coated magnetic beads (called: pellet; Supplement Fig. 1A,B). PCR was used to determine the abundance of specific DNA fragments in the sample. As a control and to normalize all samples, the same DNA sequences were amplified from samples taken before the broken DNA fragments (i.e., tagged with the biotinylated nucleotide) were recovered using the avidin-coated beads (called: input; Supplement Fig. 1A,B).

DNA of 5 × 10^8^ cells in an early-logarithmic growth phase (about 1 × 10^7^ cells/ml) was purified with the MasterPure™ purification kit (Epicentre, Madison, WI). The TdT reaction using terminal transferase (NEB, Ipswich, MA) was carried out in the presence or absence (negative control) of Biotin-14-dCTP (Invitrogen, Carlsbad, CA). DNA was precipitated and then digested in a 50 µl volume with the restriction enzyme *Bgl*II (NEB, Ipswich, MA). A small aliquot (3 µl) was removed, which was diluted 1:100 in 1xPBS and served for the “Input” PCR. The rest was diluted with 1 ml 1xPBS and 10 µl avidin-coated magnetic beads (NEB, Ipswich, MA; washed with 1xPBS) were added, which removed the broken DNA fragments with the biotinylated nucleotide extension. The DNA solution was incubated with the beads for 2 h at 4 °C. To remove nonspecifically bound DNA fragments, the beads were washed with a buffer containing 5 mM Tris–HCl (pH 8.0), 0.5 mM EDTA (pH 8.0), 1 M NaCl, and 0.05% TWEEN 20 at 50 °C for 2 h. Afterwards the beads were washed with 1 ml 1xPBS three times and resuspended in 100 µl 1xPBS (called “Pellet”). PCR at the indicated locations (see list of used primer-sets in the Supplemental Experimental Procedures) were carried out with “Pellet” and “Input” DNA. The PCR condition was 23 cycles (for rDNA) and 32 cycles (for non-rDNA locations) of denaturation at 94 °C for 30 s, annealing at 60 °C for 45 s (for rDNA) and 50 °C for 45 s (for non-rDNA locations), and elongation at 68 °C for 165 s. PCR fragments were analyzed on an 1% agarose gel and DNA bands were visualized on a Bio-Rad™ Laboratories imaging system and the intensity of the bands was determined by using the program ImageLab (Bio-Rad™ Laboratories, Hercules, CA).

### Analysis of DNA replication intermediates and determination of ERCs

DNA for visualizing DNA replication intermediates in yeast was prepared essentially as described previously^79^. Cells were grown to an early-logarithmic growth phase (about 1 × 10^7^ cells/ml). After harvesting the cells and washing with water, the cells were resuspended in nuclear isolation buffer (NIB: 50 mM MOPS free acid; 150 mM KOAc; 2 mM MgCl_2_; 150 µM Spermine; 500 µM Spermidine; 17% (v/v) Glycerol; pH 7.2) and lysed using glass beads (about 10 times for 30 s on a vortex in the cold room; cooling in between on ice) as described previously^79^. Glass beads were removed and the nuclei, broken and unbroken cells were harvested, the pellet resuspended in 4.2 ml TEN buffer (50 mM Tris–HCl, pH 8.0; 50 mM EDTA; 100 mM NaCl), Proteinase K (75 µl of a 20 mg/ml solution) and N-lauroylsarcosine (0.8 ml of a 7.5% solution). Samples were incubated at 37 °C for one hour. Non-lysed cells were removed by centrifugation in a microcentrifuge (10 min; 14,000 rpm; 4 °C). Supernatant liquids were combined and CsCl (final concentration: 1.05 g/ml) and Hoechst Dye #33258 (0.025 volumes of 5 mg/ml) were added. DNA was prepared by CsCl-gradient centrifugation (VTi65.2 rotor; 16–20 h, 55,000 rpm, 17 °C). Highly intact whole genomic DNA was digested for about 5 h at 37 °C with the appropriate restriction enzymes (*Bgl*II: rDNA; *Eco*RV: *ARS313/314*; *Bgl*II: *ARS608*; *Eco*RV: *tRNA*^Y^; NEB, Ipswich, MA)^26–28^. The structure of DNA replication intermediates were determined by neutral–neutral DNA 2D agarose gel electrophoresis and Southern technique using radioactive-labeled probes^26,27,79^. Typically, the first dimension was performed in 0.35% (w/v) 1xTBE (Tris–borate-EDTA) agarose gels at 0.6–0.7 V/cm for 45–48 h at room temperature. The second dimension was performed in 0.9% (w/v) 1xTBE agarose gels in the presence of 0.3 μg/ml ethidium bromide at 2.6–3 V/cm for 18–24 h at 4 °C. Primer sequences for the PCR amplifications of DNA probes are listed under Supplemental Information. The amounts of specific replication intermediates were determined by phosphor imager analysis.

The amount of extrachromosomal rDNA circles (ERCs) was determined by 2D gels (0.5 μg/ml ethidium bromide in both dimensions)^27^.

### Staining procedures

Yeast cells were grown to an early-logarithmic growth phase (about 1 × 10^7^ cells/ml) in the appropriate medium containing 2% or 0.05% glucose. Dead yeast cells were stained with propidium iodide (PI; final concentration: 0.3 µg/ml) in the growth medium for ~ 20 min at room temperature. PI-stained cells were visualized by epifluorescence microscopy (excitation: 493 nm). Living cells were detected with fluorescein diacetate (FDA; 5 mg/ml in acetone) as previously described^84^. FDA-stained cells were visualized by epifluorescence microscopy (excitation: 488 nm).

Staining with Singlet Oxygen Sensor Green (SOSG, Invitrogen/Molecular Probes; dilution 1:1000) was carried out on live cells together with PI. SOSG-stained cells were visualized by epifluorescence microscopy (excitation: 488 nm). Staining with MitoTracker® Red CMXRos (Invitrogen/Molecular Probes, Eugene, OR) (excitation: 493 nm) was carried out on live cells together with SOSG.

The numbers of cells on the images were determined using the software NIH imageJ^128^.

### Cell sorting

Yeast cells were grown to an early logarithmic growth phase, PI was added to a final concentration of 0.3 µg/ml. PI-stained cells were separated from unstained cells using a BD FACSAria II cell sorter with excitation off the 561 nm laser and acquisition with the PI filter set in the low 600 nm region. PI-stained and non-stained cells were collected. To determine cell viability, equal cell numbers were plated on YEPD plates (2% glucose) and incubated at 30 °C for 3 days. An aliquot of the cells was used to determine DNA breakage as described above for the TdT assay.

### Fluorescent activated cell sorting (FACS)

Yeast cells were fixed with formaldehyde (final concentration: 3.7%; early logarithmic growth phase (about 1 × 10^7^ cells/ml)) for one hour at room temperature. Cells were washed two times with 50 mM sodium citrate buffer. Cells were stained by adding PI (final concentration: 25 µg/ml) for one hour at room temperature after digestion of RNA with RNase A (final concentration: 5 µg/ml) overnight at 37 °C. The distribution of cells in G1, S and G2/M were modelled according to the ModFit LT program.

### Rad53 western blotting

Yeast cells were grown to an early-logarithmic growth phase (about 1 × 10^7^ cells/ml) in the appropriate medium containing 2% or 0.05% glucose. About 2 × 10^7^ cells were harvested, resuspended in 150 µl lysis-solution (1.85 M NaOH, 2% beta-mercapto-ethanol) and incubated on ice for 10 min. 150 µl of 50% Tri-chlor-acid were added, the solution mixed and incubated on ice for 30 min. Proteins were recovered by centrifugation (10 min, 14,000 rpm, 4 °C). Pellet was resuspended in 50 µl of protein sample buffer (containing 1 M Tris–HCl, pH8.0). Samples were heated at 70 °C for 5 min. 10 µl of each sample were loaded on an 6% SDS-poly-acryl-amide-gel and proteins transferred to nitrocellulose. Rad53 protein was detected with a rabbit polyclonal anti-Rad53 antibody (1: 2000; abcam, Cambridge, MA) followed by a horse-radish-peroxidase-conjugated secondary antibody (1:3000; Bio-Rad™ Laboratories, Hercules, CA).

### Statistics

Experiments were carried out two to three times as indicated. Standard errors were calculated.

### Supplementary Information

Supplementary Information.

## Data Availability

Further information and requests for resources and reagents as well as details about data should be directed to and will be fulfilled by the Lead Contact, Andreas Ivessa (ivessaan@njms.rutgers.edu). All unique strains and reagents generated in this study are available from the Lead Contact without restriction. Requests for strains and reagents donated by other laboratories should be directed to the specific laboratory from which they were received.
