# Supplementary material for: The increase in cell death rates in caloric restricted cells of the yeast helicase mutant rrm3 is Sir complex dependent
Source: Sci Rep. 2023 Oct 19;13:17832. doi: 10.1038/s41598-023-45125-z (PMC10587150; doi:10.1038/s41598-023-45125-z)
Supplement: Supplementary file 1 — Supplementary Information. [file 41598_2023_45125_MOESM1_ESM.pdf]

Supplemental Information

The increase in cell death rates in caloric restricted cells of the yeast helicase mutant *rrm3* is Sir complex dependent

Andreas S. Ivessa[1,3] and Sukhwinder Singh[2]

[1]Department of Cell Biology and Molecular Medicine
[2]Pathology and Laboratory Medicine/Flow Cytometry and Immunology Core Laboratory
Rutgers New Jersey Medical School
Rutgers Biomedical and Health Sciences
185 South Orange Avenue
Newark, NJ 07101-1709
U.S.A.

[3]Corresponding Author and Lead Contact:
Email: ivessaan@njms.rutgers.edu

This section includes Supplemental Results (five figures and figure legends), tables for primer sequences (Table S1), used yeast strains (Table S2) and materials (Table S3), uncropped images for PCR-gels (for Figures 1 and 3) and western-blots (for Figures 4 and 5) (other images are displayed in their entire size).

Supplement Figure 1: Control experiment for determining the DNA breakage rate by the terminal deoxynucleotidyl transferase (TdT) assay combined with PCR and schema of ribosomal DNA (rDNA)

A. Schema of determining the DNA breakage rate by the terminal deoxynucleotidyl transferase (TdT) assay combined with PCR. In the top panel, the DNA was cut with the restriction enzyme *after* extending the DNA breaks with the biotinylated-dCTP tags, thus here only natural DNA breaks are detected. In the bottom panel, the DNA was cut with the restriction enzyme *prior* to extending the DNA breaks with the biotinylated-dCTP tags, thus here natural *and* artificially created DNA breaks are detected. **B.** Agarose gels displaying the PCR products for fragment *YFH1* (see Figure 1) for WT yeast cells. (-) and (+) biotin-dCTP refer to “no addition” or “addition” of biotin-dCTP to the TdT reaction. The intensity of the bands in the gels was determined by using the program ImageLab (Bio-Rad™ Laboratories, Hercules, CA). Each pellet value was first normalized by the input value, then the (-)-biotin-dCTP value (i.e., background) was subtracted from the (+)-biotin-dCTP value. Experiments were carried out three times. Standard errors are shown. The conclusion of the control experiment is that the DNA breakage rates are higher in the samples which were digested with the restriction enzyme *prior* to extending the DNA breaks with the biotinylated-dCTP tags, thus an increased number of dsDNA breaks were present. **C.** Schema of ribosomal DNA (rDNA). The regions of fragment #1 and #2 in rDNA are indicated which were amplified by PCR (see Figure 1). The 9.1 kbp single repeat can be split into two equal parts by digestion with *Bgl*II (Bgl A and B). *ARS*: *a*utonomous *r*eplicative *s*equence; RFB: replication fork barrier.

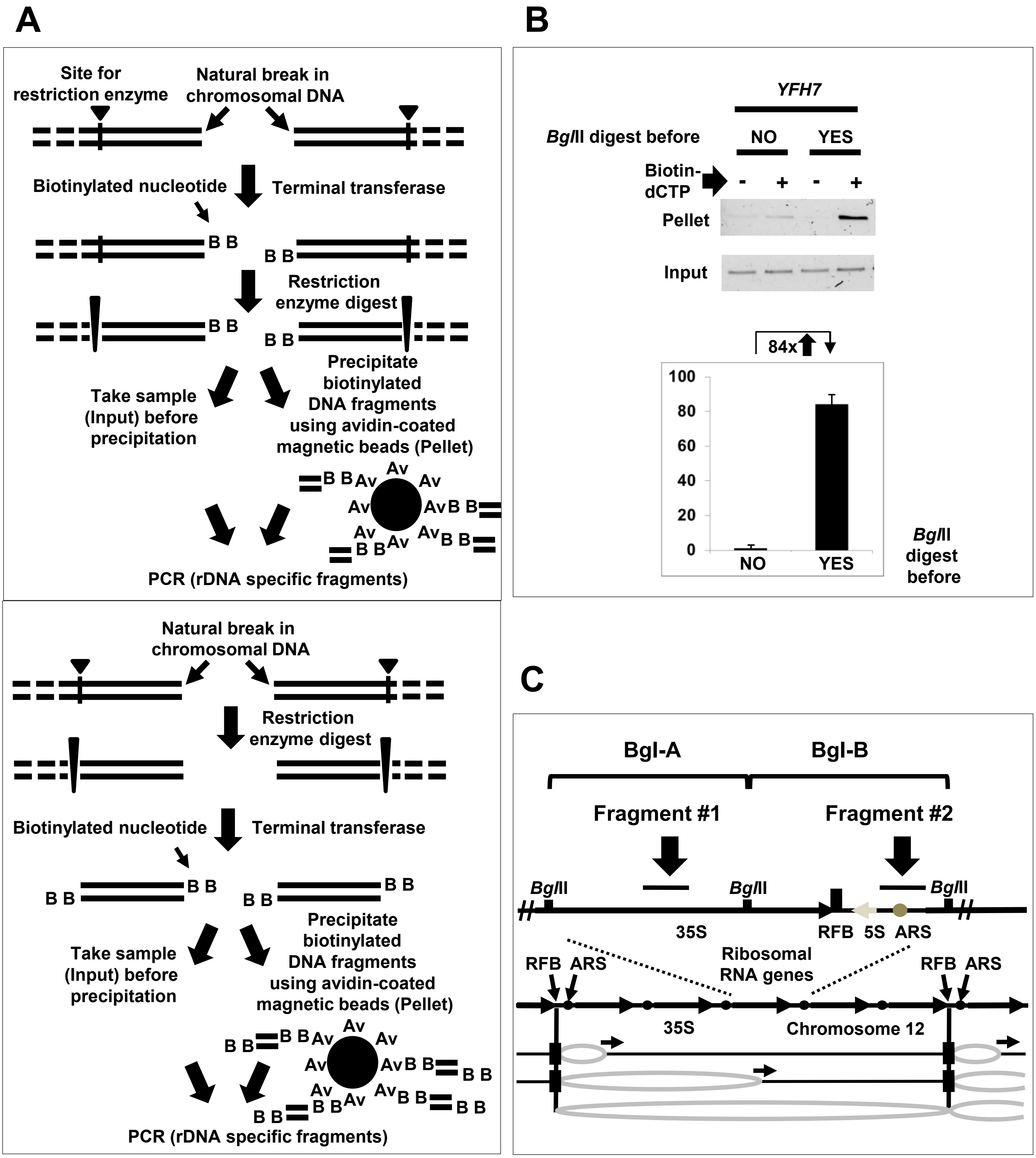

Supplement Figure 1
Ivessa and Singh

Supplement Figure 2: Numbers of propidium iodide (PI)-stained *rrm3* mutant cells (indicating dying or dead cells) are low under normal growth conditions (2% glucose) and increased under calorie-restricted growth conditions

Cells of the indicated strains were grown in medium containing 2% or 0.05% glucose to an early logarithmic phase (OD_{600} ~0.1). Representative microscopic images display PI-stained cells (orange) and the corresponding brightfield images (PI-staining superimposed). Bar: 50µm. Used strains are (in parenthesis are the approximate average cell numbers in the displayed images as determined by the program imageJ [1]): WT, pRS315 (2%: 1350; CR: 1300); *rrm3*, pRS315-*RRM3* (2%: 1400; CR: 1100); *rrm3*, pRS315 (2%: 1300; CR: 1500); *rrm3*, pRS315-*rrm3* (K260A) (2%: 1200; CR: 1400); *rrm3*, pRS315-*rrm3* (K260R) (2%: 1400; CR: 1900). The two alleles *rrm3* (K260A) and *rrm3* (K260R) are properly expressed as previously demonstrated [2]. The histogram of the percentage of PI-stained cells is displayed in Figure 3A. Experiments were carried out three times.

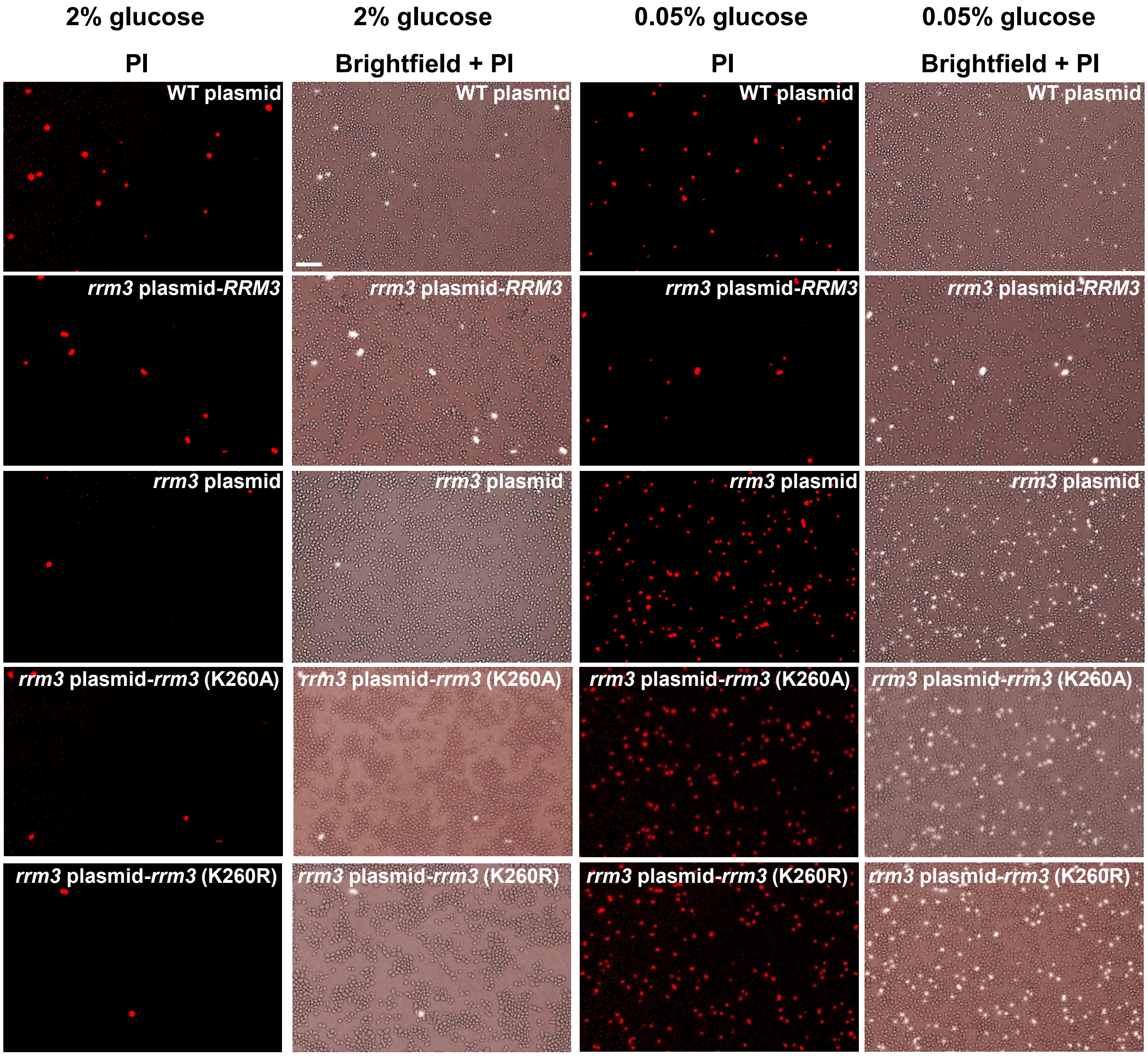

Supplement Figure 2
Ivessa and Singh

Supplement Figure 3: Whereas the absence of Pif1, Rad52 or Hdf1 has no influence on the increase in the number of PI-stained cells of *rrm3* mutant cells, which are transferred from growth medium containing 2% glucose to CR-medium, the absence of Hxk2 has a partial effect.

Cells of the indicated strains were grown in medium containing 2% or 0.05% glucose to an early logarithmic phase (OD_{600} ~0.1). Representative microscopic images display PI-stained cells (orange) and the corresponding brightfield images (PI-staining superimposed (except for *hxk2* and *hxk2 rrm3*)). Bar: 50µm. Used strains are (in parenthesis are the approximate average cell numbers in the displayed images as determined by the program imageJ [1]): WT (2%: 1000; CR: 800), *rrm3* (2%: 900; CR: 950), *pif1*-m2 (2%: 900; CR: 650), *pif1*-m2 *rrm3* (2%: 700; CR: 600), *rad52* (2%: 500; CR: 300), *rad52 rrm3* (2%: 450; CR: 400), *hdf1* (2%: 1300; CR: 480), *hdf1 rrm3* (2%: 1000; CR: 900), *hxk2* (2%: 900; CR: 800), *hxk2 rrm3* (2%: 800; CR: 800). The histogram displaying percentage of PI-stained cells is shown in Figure 3E. Experiments were carried out three times.

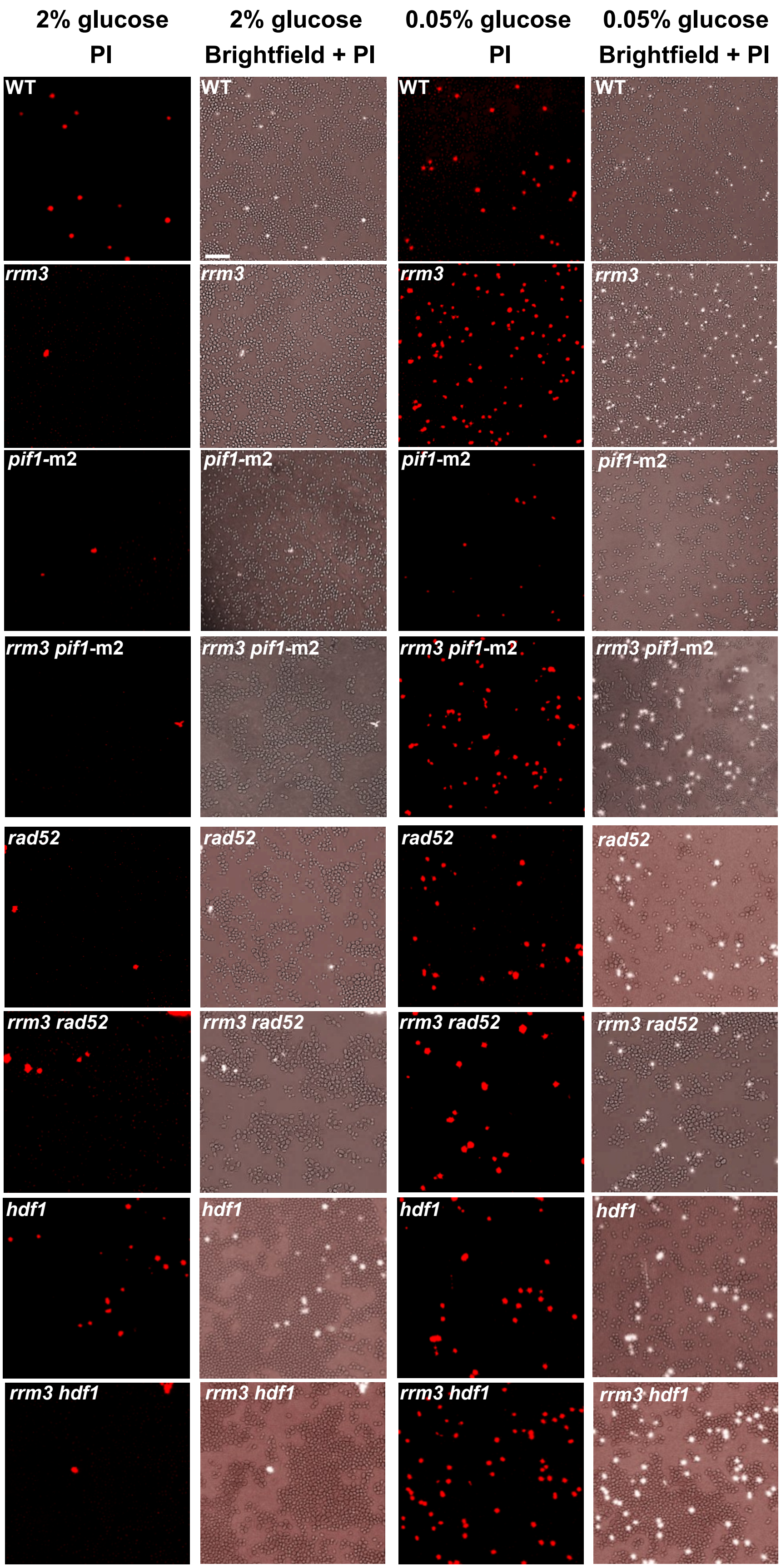

Supplement Figure 3
Ivessa and Singh

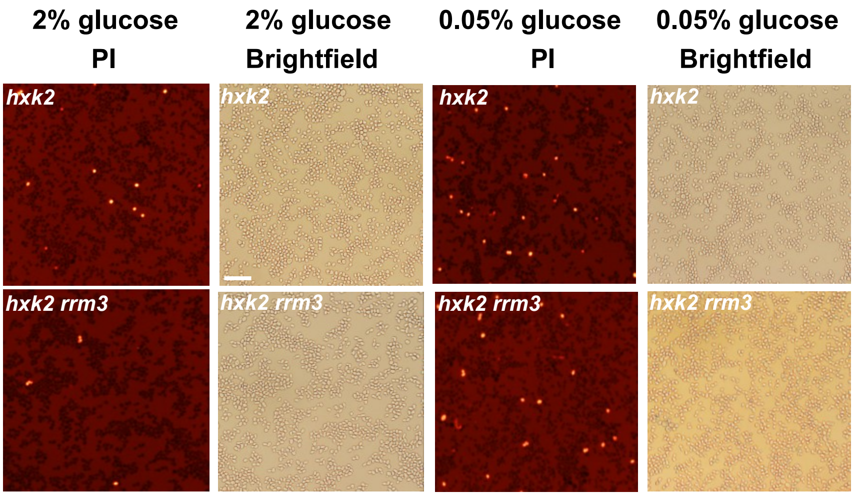

Supplement Figure 3 (continued)
Ivessa and Singh

Supplement Figure 4: The Sir2/Sir3/Sir4 complex is needed for the increase of the number of PI-stained cells of CR-*rrm3* mutant cells

Cells of the indicated strains were grown in medium containing 2% or 0.05% glucose to an early logarithmic phase (OD_{600} ~0.1). Representative microscopic images display PI-stained cells (orange) and the corresponding brightfield images (PI-staining superimposed). Bar: 50µm. Used strains are (in parenthesis are the approximate average cell numbers in the displayed images as determined by the program imageJ [1]): WT (2%: 750; CR: 650), *rrm3* (2%: 650; CR: 600), *sir2* (2%: 450; CR: 700), *sir2 rrm3* (2%: 450; CR: 450), *sir3* (2%: 500; CR: 500), *sir3 rrm3* (2%: 700; CR: 450), *sir4* (2%: 500; CR: 450), *sir4 rrm3* (2%: 600; CR: 700). The histogram displaying percentage of PI-stained cells is shown in Figure 5A. Experiments were carried out three times.

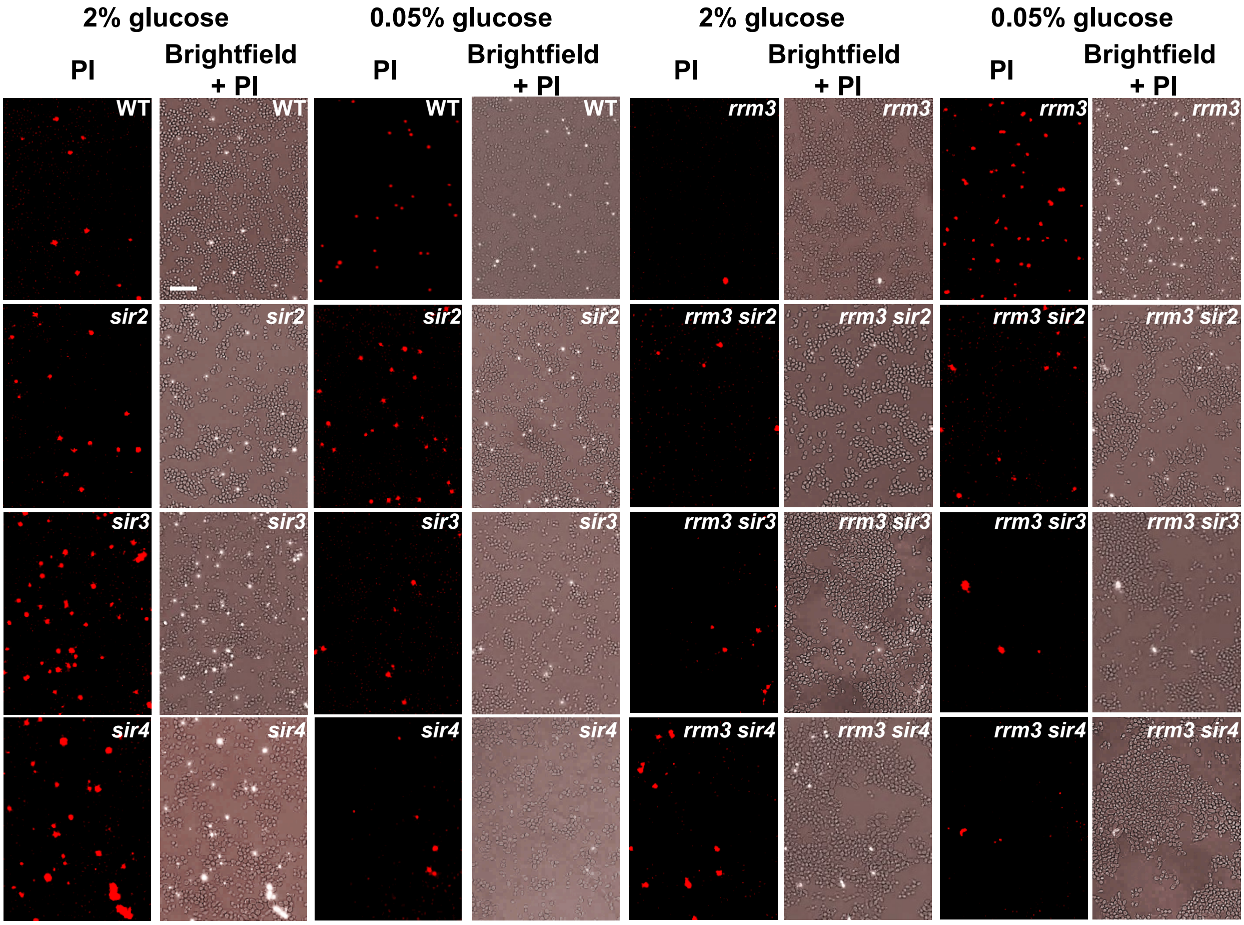

Supplement Figure 4
Ivessa and Singh

Supplement Figure 5: Reduction of mitochondrial oxidative stress lowers cell death rates in CR-*rrm3* cell populations

A. Microscopic images display double-stained cells with propidium iodide (PI; orange) and Singlet Oxygen Sensor Green (SOSG; green) with superposition of the brightfield image. Red asterisks indicate cells, which are SOSG-positive, but PI-negative. Bar: 50µm.

B. Microscopic images of the indicated strains grown in medium containing 2% or 0.05% (CR) glucose display double-stained cells with Singlet Oxygen Sensor Green (SOSG; green) and MitoTracker Red CMXRos (red). Bar: 50µm.

C. *rrm3* mutant cells were grown under CR conditions in the absence or presence of the mitochondrial antioxidant Mito-TEMPO (160 µM final concentration). Microscopic images display propidium iodide (PI) stained cells (orange) and Singlet Oxygen Sensor Green (SOSG; green) with superposition of the brightfield image. Bar: 50µm.

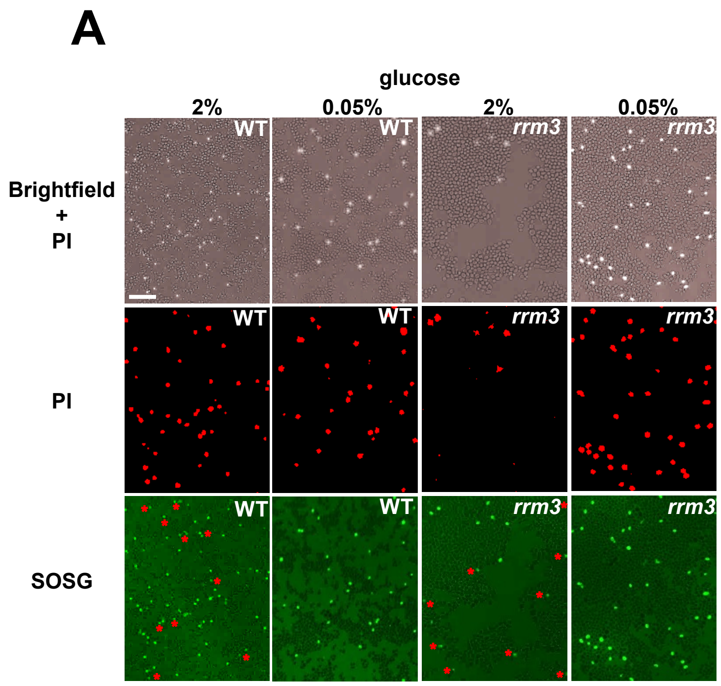

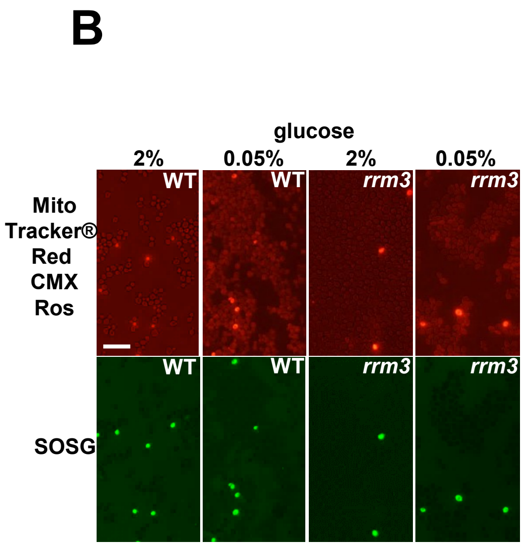

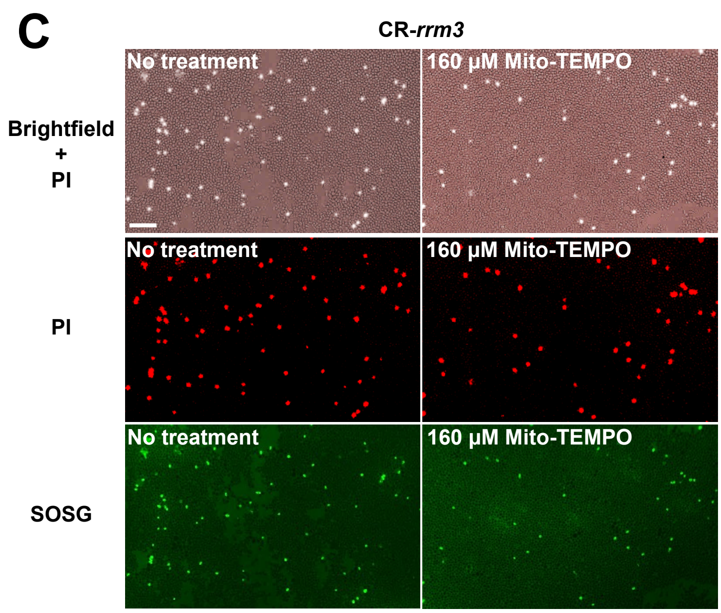

Supplement Figure 5
Ivessa and Singh

References

1 Schneider, C. A., Rasband, W. S. & Eliceiri, K. W. NIH Image to ImageJ: 25 years of image analysis. *Nat Methods* **9**, 671-675, doi:10.1038/nmeth.2089 (2012).

2 Ivessa, A. S., Zhou, J.-Q. & Zakian, V. A. The *Saccharomyces* Pif1p DNA helicase and the highly related Rrm3p have opposite effects on replication fork progression in ribosomal DNA. *Cell* **100**, 479–489 (2000).

Uncropped PCR agarose gel images for Figure 1

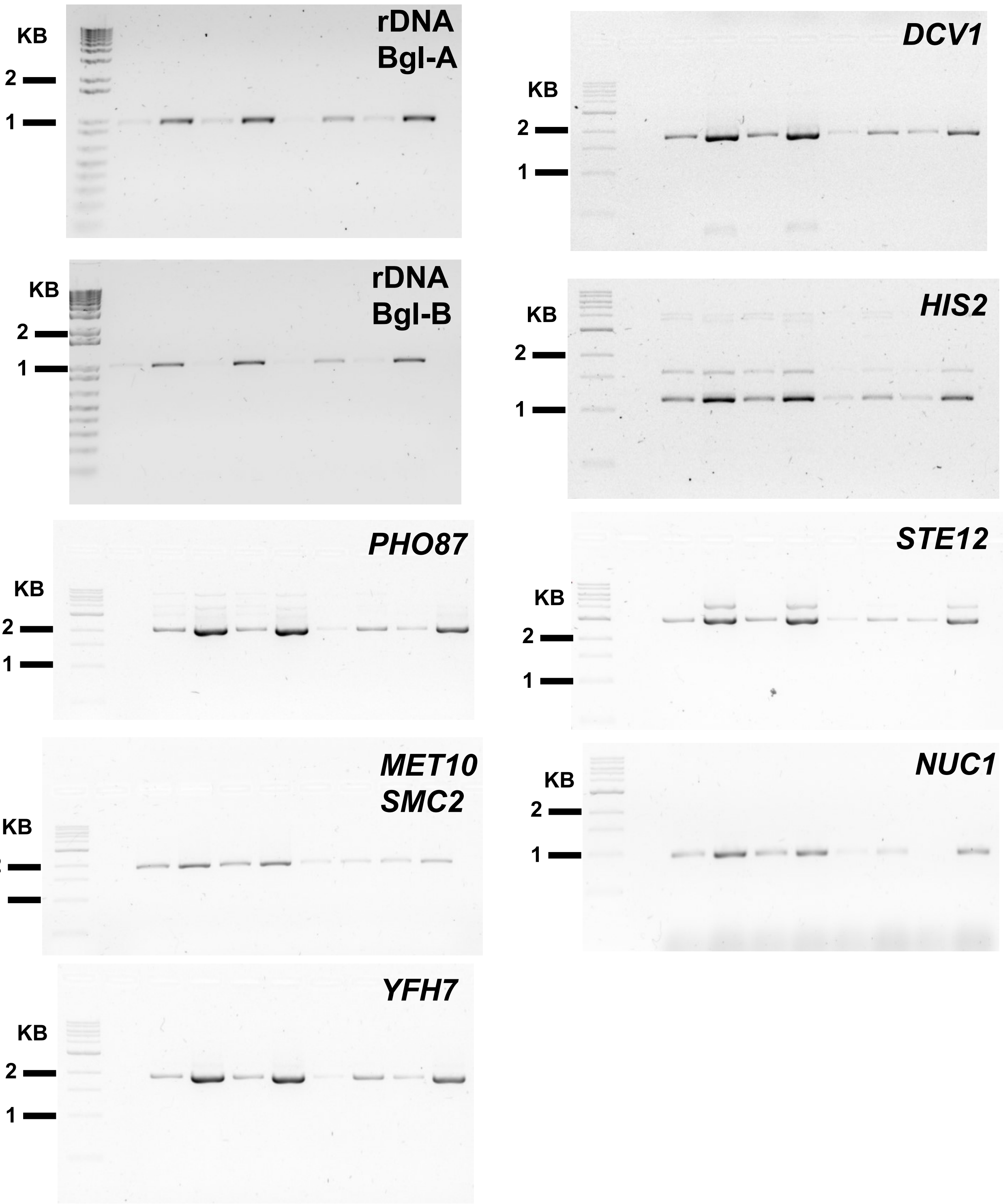

Uncropped PCR agarose gel images for Figures 1 and 3

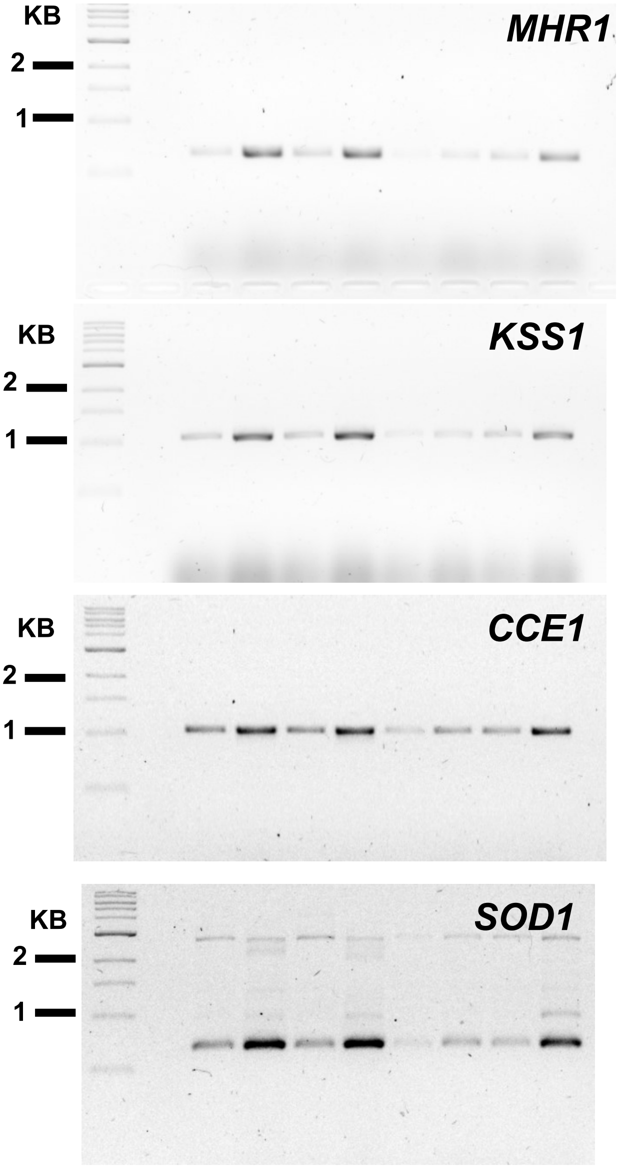

Determination of DNA breakage in sorted cells (PI-staining; Figure 3)

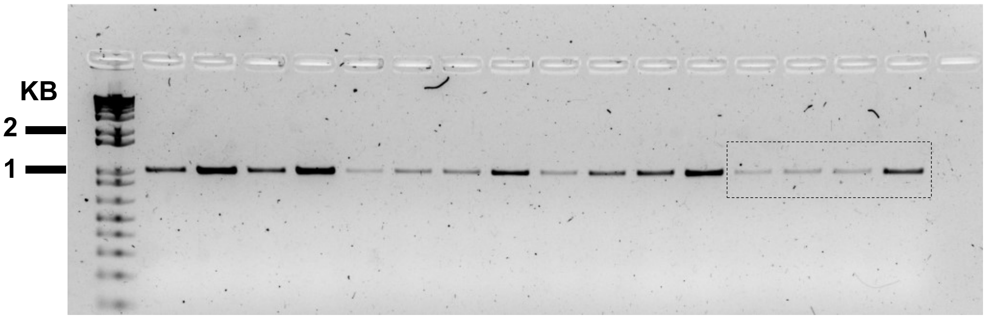

Uncropped western blot images for Figure 4B

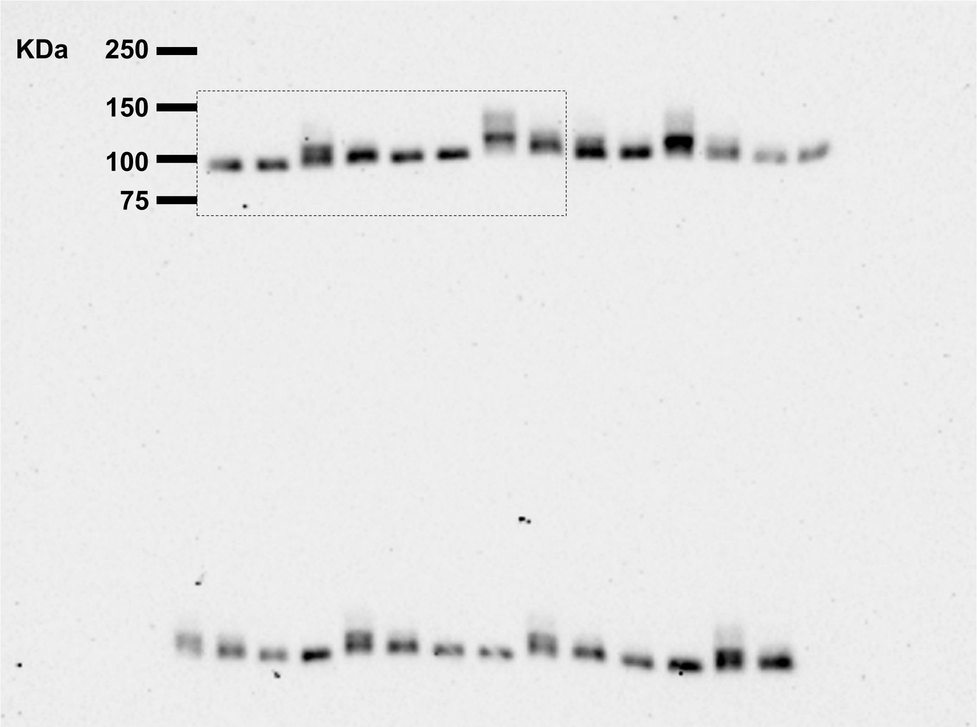

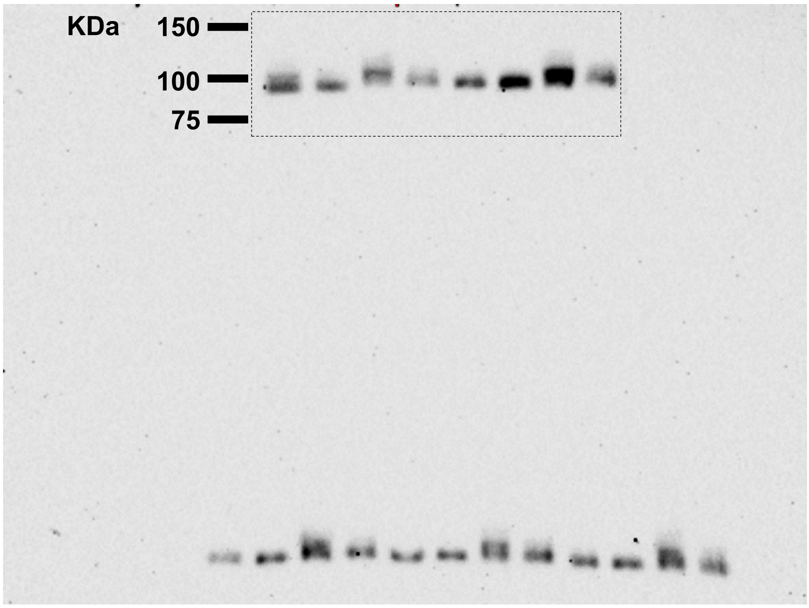

Uncropped western blot images for Figure 4C

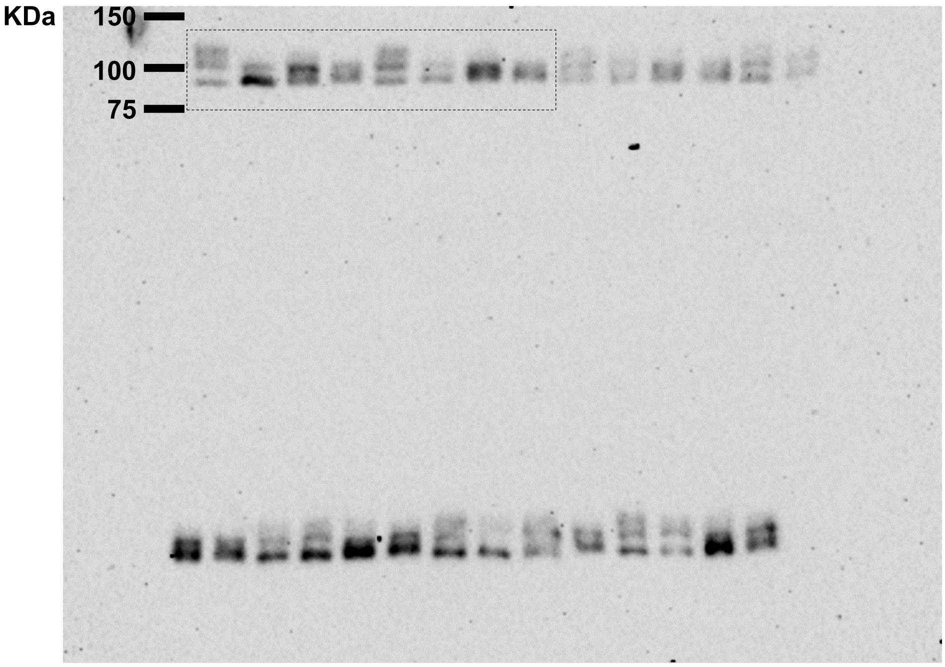

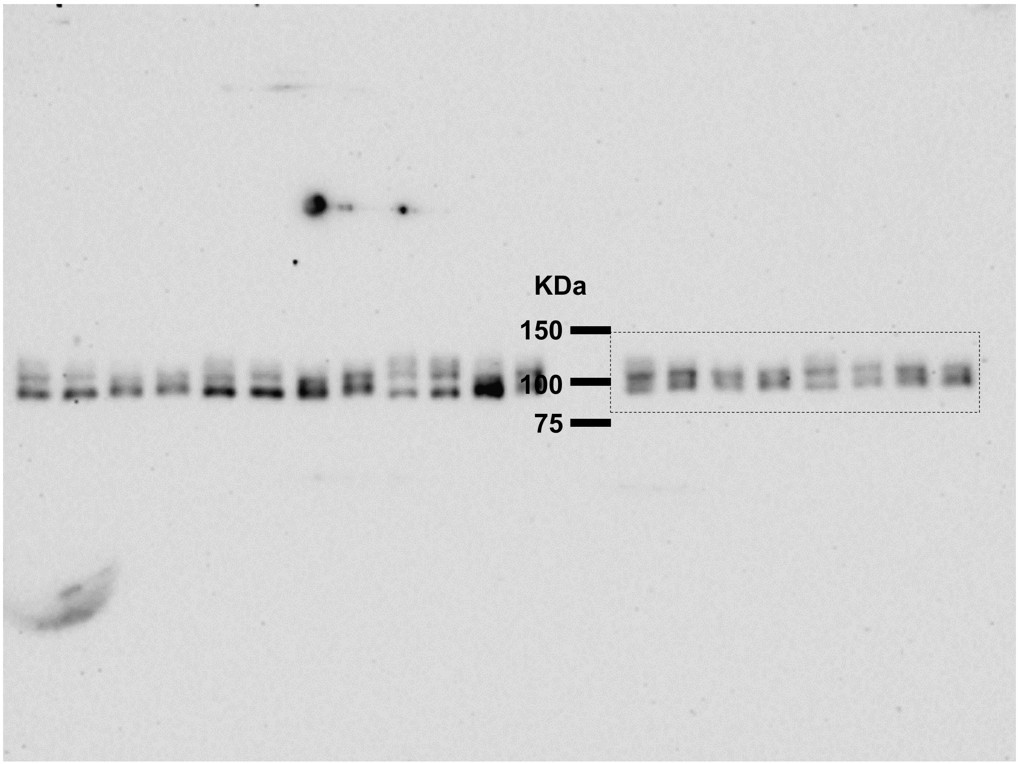

Uncropped western blot images for Figure 4D

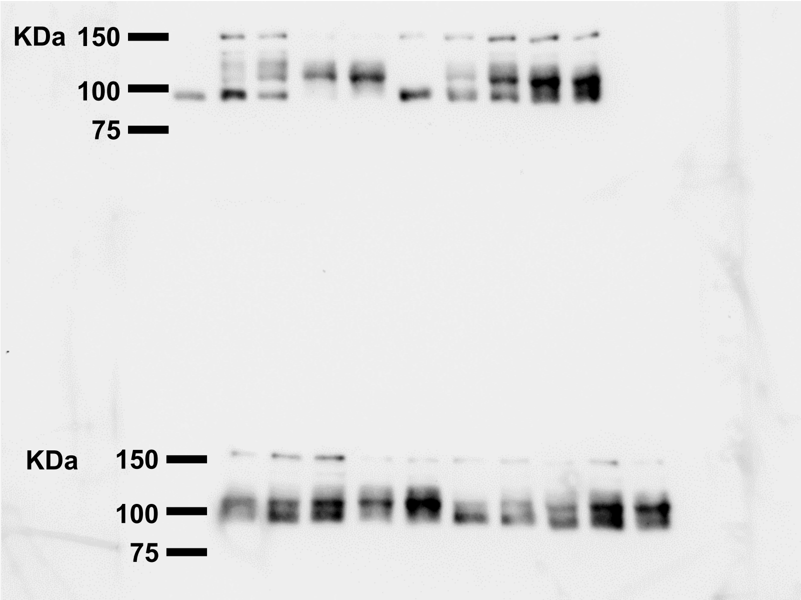

Uncropped western blot images for Figure 5C and 5D

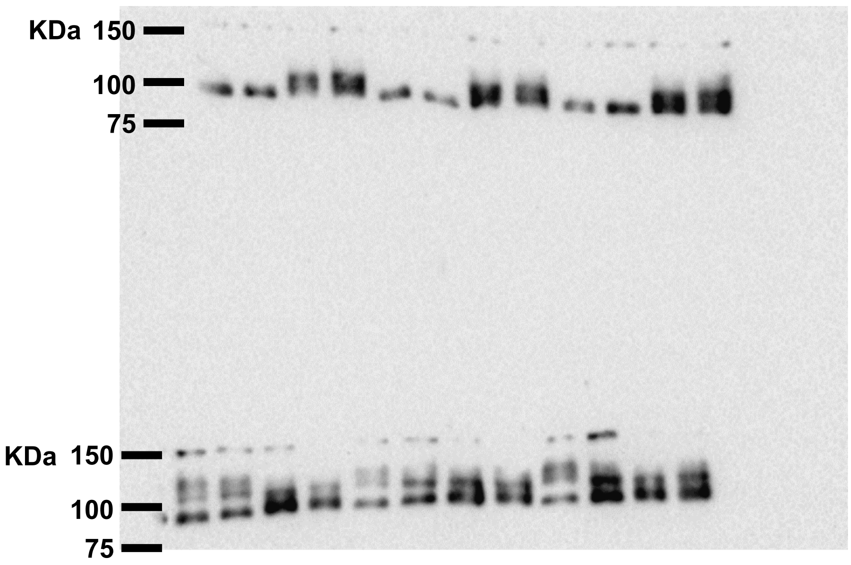

Table S1: Yeast strains used in this study

Name	Genotype	Source
VPS106 *WT*	*MATa ade2 ade3 leu2-3,112 ura3Δ trp1Δ lys2-801 can1*	Schulz and Zakian, Cell, 1994 , Vol. 76: 145
VPS106 *rrm3*	*MATa ade2 ade3 leu2-3,112 ura3Δ trp1Δ lys2-801 can1 rrm3::TRP1*	Ivessa et al., Cell, 2000, Vol.100: 479
VPS106 *pif1-* m2	*MATa ade2 ade3 leu2-3,112 ura3Δ trp1Δ lys2-801 can1 pif1-*m2	Ivessa et al., Cell, 2000, Vol.100: 479
VPS106 *rrm3 pif1-* m2	*MATa ade2 ade3 leu2-3,112 ura3Δ trp1Δ lys2-801 can1 rrm3::TRP1 pif1-*m2	Ivessa et al., Cell, 2000, Vol.100: 479
VPS106 *hdf1*	*MATa ade2 ade3 leu2-3,112 ura3Δ trp1Δ lys2-801 can1 hdf1::LEU2*	Ivessa et al., Mol. Cell, 2003, Vol.12: 1525
VPS106 *rrm3 hdf1*	*MATa ade2 ade3 leu2-3,112 ura3Δ trp1Δ lys2-801 can1 rrm3::TRP1 hdf1::LEU2*	Ivessa et al., Mol. Cell, 2003, Vol.12: 1525
VPS106 *rad52*	*MATa ade2 ade3 leu2-3,112 ura3Δ trp1Δ lys2-801 can1 rad52::LEU2*	Ivessa et al., Cell, 2000, Vol.100: 479
VPS106 *rrm3 rad52*	*MATa ade2 ade3 leu2-3,112 ura3Δ trp1Δ lys2-801 can1 rrm3::TRP1 rad52::LEU2*	Ivessa et al., Cell, 2000, Vol.100: 479
VPS106 *sir2*	*MATa ade2 ade3 leu2-3,112 ura3Δ trp1Δ lys2-801 can1 sir2::LEU2*	Ivessa et al., Mol. Cell, 2003, Vol.12: 1525
VPS106 *rrm3 sir2*	*MATa ade2 ade3 leu2-3,112 ura3Δ trp1Δ lys2-801 can1 rrm3::TRP1 sir2::LEU2*	Ivessa et al., Mol. Cell, 2003, Vol.12: 1525
VPS106 *sir3*	*MATa ade2 ade3 leu2-3,112 ura3Δ trp1Δ lys2-801 can1 sir3::LEU2*	Ivessa et al., Mol. Cell, 2003, Vol.12: 1525
VPS106 *rrm3 sir3*	*MATa ade2 ade3 leu2-3,112 ura3Δ trp1Δ lys2-801 can1 rrm3::TRP1 sir3::LEU2*	Ivessa et al., Mol. Cell, 2003, Vol.12: 1525
VPS106 *sir4*	*MATa ade2 ade3 leu2-3,112 ura3Δ trp1Δ lys2-801 can1 sir4::LEU2*	Ivessa et al., Mol. Cell, 2003, Vol.12: 1525
VPS106 *rrm3 sir4*	*MATa ade2 ade3 leu2-3,112 ura3Δ trp1Δ lys2-801 can1 rrm4::TRP1 sir2::LEU2*	Ivessa et al., Mol. Cell, 2003, Vol.12: 1525
VPS106 *WT* pRS315	*MATa ade2 ade3 leu2-3,112 ura3Δ trp1Δ lys2-801 can1* pRS315	Ivessa et al., Cell, 2000, Vol.100: 479
VPS106 *rrm3* pRS315	*MATa ade2 ade3 leu2-3,112 ura3Δ trp1Δ lys2-801 can1 rrm3::TRP1* pRS315	Ivessa et al., Cell, 2000, Vol.100: 479
VPS106 *rrm3* pRS-315-*RRM3*	*MATa ade2 ade3 leu2-3,112 ura3Δ trp1Δ lys2-801 can1 rrm3::TRP1* pRS315-*RRM3*	Ivessa et al., Cell, 2000, Vol.100: 479
VPS106 *rrm3* pRS315-*rrm3-* K260A	*MATa ade2 ade3 leu2-3,112 ura3Δ trp1Δ lys2-801 can1 rrm3::TRP1 pRS315-rrm3-*K260A	Ivessa et al., Cell, 2000, Vol.100: 479
VPS106 *rrm3* pRS315-rrm3-K260R	*MATa ade2 ade3 leu2-3,112 ura3Δ trp1Δ lys2-801 can1 rrm3::TRP1* pRS315-*rrm3-* K260R	Ivessa et al., Cell, 2000, Vol.100: 479
VPS106 *hxk2*	*MATa ade2 ade3 leu2-3,112 ura3Δ trp1Δ lys2-801 can1 hxk2::LEU2*	this study
VPS106 *rrm3 hxk2*	*MATa ade2 ade3 leu2-3,112 ura3Δ trp1Δ lys2-801 can1 rrm3::TRP1 hxk2::LEU2*	this study

Table S2: Oligonucleotides used in this study, related to the Key Resources Table

Name	Sequence
Primer sequences (Southern blots; Figure 2):	
Primer sequences for rDNA probe (between BglII and StuI):	
RDN-BglII-F	5’- CTC TCT CCA CCG TTT GAC
RDN-StuI-R	5’- AGA GAA GTA GAC TGA ACA AGT CTC
Primer sequences for ARS313/314 area and ARS608:	
ARS313-F	5’- CAG GTT TGT TTC ATT CAG CGT ATC ATC
ARS313-R	5’- TCG GTT ATG GTA TTA GTT CTT CCG TC
ARS314-F	5’- ATT ACG CAG ACA AAC GCT AAC AAC G
ARS314-R	5’- GAA ATA GGT TTG ATT ACA GCG AAG AAG
ARS608-F1	5’- TGT ACC AAC CGT ATC TGG AAA AGC
ARS608-R1	5’- CTA ATA GGG TCT TCA GGA CGA G
Primer sequences for tRNA-Y: tY[GUA]F1	
tRNA_Y-F1	5’- GCA TTG ATT ATT TAG GAA GCA CCT C
tRNA_Y-R1	5’- GGA CTT ACA GAC CAA ATC CTT TAT AC
tRNA_Y-F2	5’- TTC GTA ACA ATT CAA TGG CTT GCC
tRNA_Y-R2	5’- GGA GAT TTG ATT TCA GGC GGT G
Primer sequences (TdT assays; Figure 1):	
Primer sequences for rDNA fragments #1 and #2:	
RDN-Frag_1-F	5’- CTG TTA TTG CCT CAA ACT TCC ATC
RDN-Frag_1-R	5’- GAT GGA AGT TTG AGG CAA TAA CAG
RDN-Frag_2-F	5’- CAC CCA TAA CAC CTC TCA CTC C
RDN-Frag_2-R	5’- GGA GTG AGA GGT GTT ATG GGT G
Primer sequences for non-rDNA fragments:	
PHO87 (ARS313-F)	5’- CAG GTT TGT TTC ATT CAG CGT ATC ATC
PHO87 (ARS313-R)	5’- TCG GTT ATG GTA TTA GTT CTT CCG TC
MET10/SMC2 (ARS608-F1)	5’- TGT ACC AAC CGT ATC TGG AAA AGC
MET10/SMC2 (ARS608-R1)	5’- CTA ATA GGG TCT TCA GGA CGA G
YFH7 (tRNA-F: tF[GAA]F) F	5’- GCG AAG GCA TTG TTT ACA TGC C
YFH7 (tRNA-F: tF[GAA]F) R	5’- CCC ATC AAC GGG AGT TAC TCT G
DCV1 (tRNA-Y: tY[GUA]F1) F	5’- GCA TTG ATT ATT TAG GAA GCA CCT C
DCV1 (tRNA-Y: tY[GUA]F1) R	5’- GGA CTT ACA GAC CAA ATC CTT TAT AC
HIS2 (tRNA-A: tA[AGC]F) F	5’- CGC ATA AAG TAG TTC GGG CAG C
HIS2 (tRNA-A: tA[AGC]F) R	5’- AAG GAG AAA CAT CAA TAA GAG AAT AGT TTG
STE12-F	5’- CCT CAC ACC CCA TCC TAA ATA TCC
STE12-R	5’- CAA CTG TGT ATA TAC TTT GTC GAT AAT AAG

NUC1-F	5’- CGT TAC ATA TGT GCA GTA GGA TAC TCT TGT CC
NUC1-R	5’- CTT GAT CTC GAG ATT CCT TTT TTT TGG AGG AGG TAA CAA TTT C
MHR1-F	5’- CGT TAC ATA TGA AGG TAA ACC ATT CAA TTT CAA G
MHR1-R	5’- CTT GAT CTC GAG CTG GGA AGA CAC TTC CGT CTG
KSS1-F	5’- ATG GCT AGA ACC ATA ACT TTT GAT ATC CC
KSS1-R	5’- CTA TTC CAT GGT CTT CAT TAG TTC ATC
CCE1-F	5’- ACA GCA CAG AAA GCT AAG ATA TTG
CCE1-R	5’- TTG TAA GTG TTC TGC AAA AAT TTC AGC
SOD1-F	5’- AAG AAG ATG GTT TTG GGC AAA TGT TTA GCT G
SOD1-R	5’- AGC TCC GGC ACA CTT GGT GAA G

Knock-out of HXK2

HXK2delLEU2 F	5’- CTTTGAAAAGGTTGTAGGAATATAATTCTCCACACATAATAAGTACGCTAATTAAATAAAcgtttcggtgatgac
HXK2delLEU2 R	5’- GTTCACATAAGTAGAAAAAGGGCACCTTCTTGTTGTTCAAACTTAATTTACAAATTAAGTttcctgatgcggtattttctcct

HXK2_F	5’- CTA TAA AAA AGA GTA ATC CTA CCC CAT ATC
HXK2_R	5’- CAG TTG TAA AAG TAA GAA AAA AAA AGA TCA TAG

Table S3: Key Resource Table

**Reagent or Resource**	**Source**	**IDENTIFIER**
MasterPure™ purification kit	Epicentre, Madison, WI	MPY80200
Hoechst Dye #33258	Sigma-Aldrich, St. Louis, MO	B2883
**Antibodies**		
anti-Rad53 antibody	abcam, Cambridge, MA	ab104232
Goat Anti-Rabbit IgG (H + L)-horse radish peroxidase	Bio-Rad™ Laboratories, Hercules, CA	1706515
**Chemical, Peptides, Recombinant Proteins**		
Biotin-14-dCTP	Invitrogen, Carlsbad, CA	19518-018
Streptavidin-coated magnetic beads	NEB, Ipswich, MA	S1420S
Spermine	Sigma-Aldrich, St. Louis, MO	85590
Spermidine	Sigma-Aldrich, St. Louis, MO	S2626
Propidium iodide	Sigma-Aldrich, St. Louis, MO	P4170
Singlet Oxygen Sensor Green, SOSG	Invitrogen/Molecular Probes, Eugene, OR	S36002
MitoTracker® Red CMXRos	Invitrogen/Molecular Probes, Eugene, OR	M7512
Sirtinol	Sigma-Aldrich, St. Louis, MO	S7942
Mito-TEMPO	Sigma-Aldrich, St. Louis, MO	SML0737
Fluorescein diacetate	Sigma-Aldrich, St. Louis, MO	F7378
**Experimental Models: Organisms/Strains**		
Baker's yeast (*Saccharomyces cerevisiae*)	see attached table	
**Oligonucleotides**	see attached table	
**Software and Algorithms**		
ImageLab	Bio-Rad™ Laboratories, Hercules, CA	https://www.bio-rad.com/en-us/product/image-lab-software?ID=KRE6P5E8Z
ImageJ		https://imagej.net/ij/index.html
